# Genetic Diversity Analysis of Risk Variants Associated with Bone and Cartilage Metabolism in Nine Mexican Subpopulations

**DOI:** 10.3390/biomedicines14071470

**Published:** 2026-06-29

**Authors:** Ismael Nuño-Arana, Alejandra Villagómez Vega, Gabriela Martínez Cortés

**Affiliations:** 1 Centro de Investigaciones Multidisciplinarias en Salud, Departamento de Salud y Enfermedad, Centro Universitario de Tonalá, Universidad de Guadalajara, Tonalá 45425, Jalisco, Mexico; 2 Centro de Investigaciones Multidisciplinarias en Salud, Departamento de Ciencias Biomédicas, Centro Universitario de Tonalá, Universidad de Guadalajara, Tonalá 45425, Jalisco, Mexico; alejandra.villagomez5427@academicos.udg.mx; 3 Instituto de Investigación en Genética Molecular, Departamento de Ciencias Médicas y de la Vida, Centro Universitario de la Ciénega, Universidad de Guadalajara, Ocotlán 47810, Jalisco, Mexico; gabriela.mcortes@academicos.udg.mx

**Keywords:** allelic risk, genetic diversity, genetic risk profiles, bone and cartilage metabolism, osteoporosis genetic profile, Pro-inflammatory risk

## Abstract

**Backgrounds/Objectives:** Allele frequencies of genetic variants associated with complex diseases can contribute to varying degrees to predisposition depending on the population’s genetic profile. The aim of this study was to analyze the genetic diversity of 15 relevant SNVs that could modulate bone and cartilage metabolism in underrepresented structured populations. **Methods:** In a sample of 130 Mestizos and 304 natives from 8 native Mexican populations, SNVs related to multifactorial diseases were genotyped using a SNaPShot Multiplex kit and analyzed via capillary electrophoresis using an ABI PrismTM 3130 Genetic Analyzer (Applied Biosystems, Waltham, MA, USA.), and genetic profiles for 15 SNVs were obtained using GeneMapper software v. 3.2. Allele frequencies were calculated by locus and population using Power Stats and Arlequin v.3.1 software, for which the EM algorithm was used to compare reference populations obtained from the dbSNV database of the International HapMap project. Population structure, paired comparisons, and genetic differentiation between native, admixed, and reference populations (*p* value) were estimated through *Fst* tests using the STRUCTURE v.2.3.2 and Arlequin v.3.1 software. **Results:** Haplotype frequency combinations grouped as profiles showed higher predominance in the allelic combination A/A/G for rs9340799 (*ESR1*), rs700518 (*CYP19A1*), and rs1800795 (*IL6*) genes, respectively. **Conclusions:** Allelic profiles could be useful as medical tools for preventing and managing individuals or populations. Mexican populations showed high genetic variability among allelic risk profiles for estrogen control and response, as well as high frequencies of variant combinations associated with an increased inflammatory response, potentially resulting in high osteoclastogenesis. This analysis advances our understanding of the complexity of bone and cartilage metabolism in highly stratified populations.

## 1. Introduction

Genome-wide association studies (GWASs) have helped researchers identify risk loci involved in predisposition to complex diseases by highlighting associations between genetic variants, in addition to providing information about complex traits in samples from different populations [1,2]. One of the main challenges in GWASs is to identify causal genes and understand the mechanism by which they affect the phenotype [3]. The results of these studies can have various implications in the diagnosis of diseases or new therapies [4,5]. However, these studies have been predominantly conducted in populations with European and Asian ancestry, and little is known about the frequency of risk alleles in other populations, particularly Latin-Americans. Mexicans are underrepresented in public SNV databases [6], and even the most studied populations can be poorly defined; this is because some groups do not necessarily share the same biological diversity, since subgroups can occur even within the same population. This lack of information makes it difficult to translate population-based genetic studies [7] because many variants significantly associated with certain phenotypes in some ethnic groups may be absent or rare in others. Moreover, population differences in the prevalence of certain alleles could account for a certain proportion of disease trait variation between different ethnicities [4]. Many researchers have analyzed how ethnicity could provide information about frequency variations in the risk alleles of common diseases among populations, which would allow for the prediction of whether a person has a risk allele. This correlation between ethnicity and genotype has been found to be strongest if the disease-associated variants differ in frequency [8,9]. However, this model should be tested by analyzing the frequencies of these variants in populations with different ethnic backgrounds [9,10,11,12].

Many genetic variations have been detected that are associated with osteoporosis, arthritis and other diseases. On their own, their effects are negligible, but when they are linked to others, their effects can be amplified [13,14,15]. Each SNV has its own effect, but it is not entirely understood how SNVs might interact with each other between loci. Understanding combinations of SNVs could help to better define susceptibility profiles and clarify the mechanisms involved in complex diseases [16,17,18,19].

The genetic variations selected for this study were obtained from GWASs and association studies on Mexican populations with high clinical relevance, most of which focused on diseases associated with decreased bone density and cartilage inflammatory responses [11,13,19]. The study aimed to analyze the genetic diversity of disease risk allele profiles across nine different Mexican subpopulations by examining 15 SNVs associated with bone and cartilage metabolism, and to compare the frequencies with those of other populations around the world.

## 2. Materials and Methods

### 2.1. Population Samples

Genomic DNA was extracted from 600 blood samples, only 434 of which had undergone complete genotyping, including 130 Mestizos and 304 Native Americans. All participants signed an informed consent form approved by the Ethical Research Committee of the CUCiénega-UdeG. The Native American sample included individuals affiliated with Ancestral Historical Communities (according to INALI), who self-identified as members of their group, and who spoke their group’s native language and had ancestral lineage. The participants belonged to the following native groups: Mayas from Quintana Roo, Tzeltales and Lacandones from Chiapas, Mazatecas from Oaxaca, Purépechas from Michoacán, Nahuas from Jalisco, Huicholes from Jalisco and Tarahumaras from Chihuahua. The Mestizo sample consisted of individuals from the states of Aguascalientes, Chiapas, Chihuahua, Jalisco and Yucatán (CUCienega Biobank). Both groups consisted of healthy volunteers. The study was conducted in accordance with the Declaration of Helsinki and approved by the Institutional Review Boards (or Ethics Committees) of the Institute of Molecular Genetics Research, the University Center of La Ciénega, the University of Guadalajara, and the Old Civil Hospital of Guadalajara.

### 2.2. Selection of Candidate Genes

The genetic variations were obtained from GWASs and associated studies on Mexican populations that showed high clinical relevance, most of which focused on diseases associated with bone and cartilage metabolism (Table 1).

### 2.3. Genotyping

Blood samples were obtained for each individual, and genomic DNA was extracted using standard techniques. DNA concentration of the samples was adjusted to 10 µg/mL, and two multiplex PCR methods were designed to amplify 15 DNA fragments containing the 15 SNVs. The reactions were performed with the Multiplex PCR Master Mix (QIAGEN Inc., Hilden, Germany). PCR products were purified with ExoSAP-IT (Amersham Biosciences, Piscataway, NJ, USA), and the SNVs were genotyped using a SNaPShot Multiplex kit (Applied Biosystem, Foster City, CA, USA), which is based on the dideoxy single-base extension of an unlabeled oligonucleotide primer (Supplementary Materials). A polymerase extends the primer, adding a single fluorescently labeled dideoxy nucleotide (ddNTP) to its 3’end. Primers of different lengths were designed for each polymorphic site so that the sites could be discriminated in a multiplex reaction. Amplified products were analyzed by capillary electrophoresis using an ABI PrismTM 3130 Genetic Analyzer (Applied Biosystems, Waltham, MA, USA). The assay involved three phases: (1) amplification of the target sequences by multiplex PCR; (2) purification of PCR products; and (3) a minisequencing reaction (SBE), followed by the separation and detection of minisequencing products by capillary electrophoresis [32]. The results were analyzed using Genemapper 3.2 software, and genotypes were assigned according to the length of the primer oligonucleotide and the color of the incorporated ddNTP.

### 2.4. Statistical Analysis

**Figure 1:**
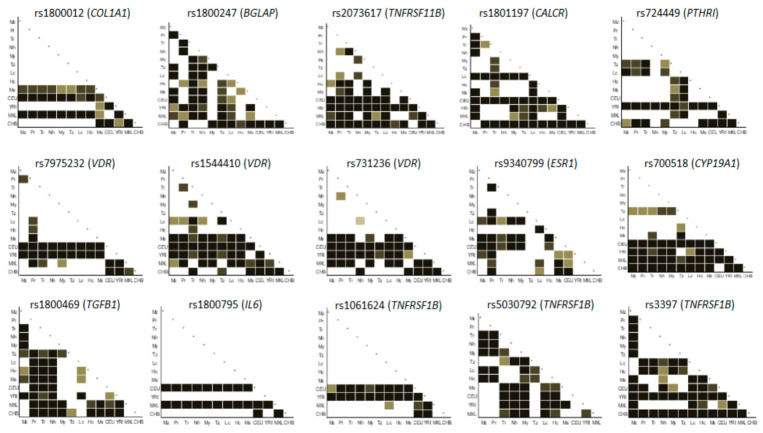
Population differentiation for 15 disease-associated SNVs. The shaded boxes in the 14 × 14 matrices show which pairwise *Fst* values are significant at three *p* value thresholds (see the incorporated *p* value legend). The title of each histogram includes the dbSNV ID and gene, the location of which is shown in the histogram.

Hardy–Weinberg equilibrium was determined using exact tests. Significance levels were assessed through 10,000 bootstrap iterations with 95% confidence intervals (IC) using Arlequin 3.1 software [33,34]. Allele and genotype frequencies were calculated by locus and population using Power Stats v1.2 [35], and allele frequencies were compared to the reported allele frequencies of the reference populations from the International HapMap project, included in the dbSNV database of NHBI (Figure 1). The data from the HapMap project represented major global populations and were compared with the results of this study. CEU consisted of samples of Utah residents with Northern and Western European ancestry from the CEPH collection (*n* = 180); CHB consisted of samples taken in Beijing from Han Chinese individuals who were unrelated to each other and with at least three or four generations of Han ancestors (n = 90); MEX consisted of individuals residing in Los Angeles, CA, U.S., with Mexican ancestry (three to four generations), with samples corresponding to parents–child pairs (*n* = 90); YRI consisted of samples taken in a Yoruba community in Ibadan, Nigeria, from parent–child pairs (*n* = 180); and JPT consisted of samples taken in the metropolitan area of Tokyo, Japan, from individuals not related to each other, with at least three generations of Japanese ancestry. A Bonferroni correction was applied based on the allele frequencies of groups with low sample numbers.

For the interpopulation comparison, the *Fst* values were estimated, and *p* values were calculated to evaluate levels of population differentiation among Mexican populations and between these and reference populations.

We analyzed the population structure of native and mixed populations with these SNVs. To minimize information loss, STRUCTURE 2.3.2. software was employed [36,37]. To run the parameters, an admixture model with a Burning Period of 10,000 iterations was computed with a probability value (Alpha) for each population. The correlation between allele frequencies was determined, where it was assumed that the results would be similar to those of the K population due to migration or common ancestry. To perform a run, the clusters (K) were set from two to six (K = 2 to 6) with a minimum of 50 iterations. For each value of K, the best run was employed with the top likelihood rate and lowest error.

To build profiles of genetic haplotypes, frequencies were calculated from the EM algorithm using the Arlequin 3.1 software, with a maximum iteration number of 1000 and a confidence interval of 95% [34], in order to obtain the allelic combinations and their population frequencies. The linkage disequilibrium (LD) between the variant haplotypes was also calculated to measure genetic diversity.

## 3. Results

We evaluated 130 Mestizos and 304 Native Americans. Table 2 presents the demographic characteristics of the included healthy volunteers.

### 3.1. The Allele Frequencies

The allele frequencies between Native American groups showed considerable variability. Only the allele frequencies of rs1800012 (*COL1A1*), rs1800795 (*IL6*), and rs700518 (*CYP19A1*) differed by less than 5%. Four SNVs (rs7975232, rs1544410, and rs731236 in the *VDR* gene and rs1061624 in *TNFRSF1B*) differed in allele frequency by 11–20%, whereas five differed by 21–30%, including rs9340799 (*ESR1*), rs2073617 (*TNFRSF11B*), rs724449 (*PTHR1*), rs5030792, and rs3397 (*TNFRSF1B*); for rs1800469 (*TGFB1*), the difference was 32%, for rs1800247 (*BGLAP*) it was 43%, and for rs1801197 (*CALCR*) it was 52%. The variants rs9340799, rs700518, and rs5030792 showed allelic frequencies higher than 65%, and rs724449 also exhibited high frequencies, ranging from 35 to 62.5% (Table 3).

After Bonferroni correction, the following SNVs deviated from Hardy–Weinberg equilibrium: rs700518 (*CYP19A1*) in Tzeltales, rs9340799 (*ESR1*) in Mazatecas, rs1800469 (*TGFB1*) in Lacandones, rs1800012 (*COL1A1*) and rs5030792 (*TNFRSF1B*) in Tarahumaras, and rs1061624 (*TNFRSF1B*) in Purépechas (Supplementary Materials). The haplotype frequencies combined as profiles showed a higher predominance in the allelic combination A/A/G for rs9340799, rs700518, and rs1800795, accounting for 56% of the total sample (Table 2). Individually, the frequencies of these SNVs were around 79.1, 98.3, and 100%, respectively, for the total populations.

Broadly, Lacandones and Tzeltales did not exhibit profiles with risk alleles, whereas Mayas and Purépechas exhibited two and three risk profiles, respectively. The Mestizo population had a similar pattern to the previous populations. Finally, Mayas and Huichol had higher haplotype frequencies, amounting to 10% (Table 4).

### 3.2. Genetic Diversity Analyses

We compared the allele frequencies of Mexican populations with those reported in the dbSNV database for the HapMap samples. The differences in allele frequencies between populations for these variants were measured using Fst analysis. Within-population differences for the 15 SNVs were not significant (*p* < 0.05) across all alleles (Nah/Tar-Lac/Pur) (Fst genetic distance range: 0.006–0.085) (Table 5). The values of each individual variant per population (Figure 1) are presented in the Supplementary Materials. In contrast, when analyzing interpopulation differences among the HapMap populations, they differed from the other non-American populations (Afr-Asian) (Fst genetic distances: 0.100–0.222; *p* < 0.001) (Table 5). However, when analyzing intrapopulation differences for each SNV, genetic differentiation was observed in *BGP* (rs1800247), *VDR* (rs1544410), *CALCR* (rs1801197), *TNFRSF1B* (rs5030792), and *TGFB1* (rs1800469) (Figure 1). The rs1061624 variant of *TNFRSF1B* showed no differences, despite having a high frequency in most populations (Figure 1) (Table 5). This suggests that an individual’s risk alleles can differ from those of others in the same ethnic group, even if their ethnic differences are not clear.

Linkage disequilibrium analysis showed the expected results for markers originating from the same gene system (*VDR* and *TNFRSF1B*) (Supplementary Materials). However, most markers, because their loci are located on different chromosomes, showed a higher recombination rate. Therefore, greater allelic variability was observed in all Mexican populations, with the exception of *COL1A1* and *IL6*, which showed low variability (less than 5%).

### 3.3. Genetic Structure Analyses

**Figure 2:**
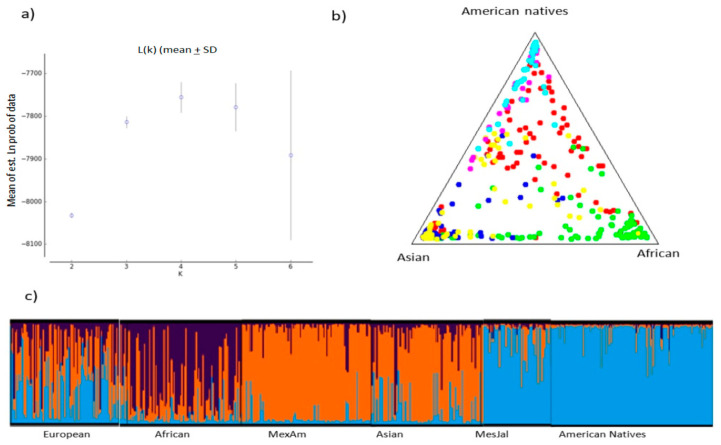
Membership proportions of each predefined population for K = 3. (**a**) Likelihood variance values. (**b**) Triangle plot for K = 3: European (red), MexAm (blue), Western Mestizo (pink) African (green), Asian (yellow). (**c**) Bar plot for 537 individuals.

Finally, the triangle plot from the ancestry analysis shows a slight tendency to separate three main components (European, Amerindian, and African, which are the three principal ancestral components of the Mestizo and native populations in Mexican history) (see Figure 2a). Similar results were obtained upon analyzing likelihood values, where the ideal clustering solution was K = 3, with the lowest standard deviation value (See Figure 2b). However, the bar plot shows that the genetic factor was not significantly different between Mestizo and indigenous groups from Mexico. Although SNVs are not usual markers for ancestry calculations, this result is consistent with the low-level differentiation in allelic frequency observed in most SNVs (Table 3 and Figure 2c).

## 4. Discussion

Public databases containing already well-characterized reference samples have continued to expand, improving our ability to estimate population allele frequencies. However, whereas Caucasian and Asian groups are widely described, groups like Latin Americans and Native Americans are underrepresented or even absent in these databases [6,38,39].

Genome-wide association studies

Knowledge of allele frequencies is fundamental to understanding risk, progression, and prognosis as biological factors in disease. GWASs have advanced our knowledge of genomic variants, particularly those of biomedical interest. Differences have been observed between populations, with some researchers suggesting that they could be risk factors for disease; as such, knowledge of the populations that represent a society is invaluable, particularly in the health field. Moreover, the knowledge obtained from these studies will be valuable for improving disease management efficiency, especially in highly heterogeneous and stratified societies. Genomic variants are currently widely studied, but scarcely from a biomedical perspective [38,40,41,42].

Most genetic susceptibility studies are conducted in European and Asian populations, and the genetic and disease information obtained from them cannot be fully extrapolated to Latin American populations due to a lack of knowledge regarding the genetic distribution of risk alleles for certain diseases.

The Mexican population has a heterogeneous genetic background. Mestizos, representing 90% of the total population, have a complex admixture of Amerindian, European, and African ancestries, like a mosaic, with ratios that vary by geographic site and social phenomena. The proportion of individuals with Amerindian ancestry is higher in the southern part of the country and decreases towards the center and the north. Demographic events like admixture and diversity of natives mean that the actual Mexican population is largely heterogeneous, genetically sub-structured, and constantly changing [43,44,45,46].

Therefore, understanding risk variants in the Mexican population, while also considering native populations, allows us to learn more about the genetic makeup of the population. Knowledge of the allele frequencies of the aforementioned variants can contribute to this endeavor. It is worth noting that these variants are associated with the Mexican population, and although their numbers are limited, valuable information can be obtained about their presence in this population.

Population structure with risk variants

In the STRUCTURE analysis of the 15 studied variants, when compared with other studies that identify a greater number of markers of population structure in these same samples, we observed a high degree of congruence in terms of genetic diversity between populations. We emphasize that this was not the objective of the study, due to the limitations imposed by bi-allelic markers, and we therefore cannot make anthropological inferences. However, it provides us with a clear overview of Mexican populations with these disease biomarkers, highlighting their substantial variability. Binary markers have lower variability than multiple-allele loci, requiring much smaller sample sizes to obtain accurate allele frequency estimates. Furthermore, most do not exhibit linkage disequilibrium because most variants are located at different loci, which increases diversity (Supplementary Materials) [13,41,42,43,44,45,46,47,48].

It has previously been shown that, on average, local positive selection has had no widespread effect on disease allele frequency differences between populations, and the allele frequency differences in disease-associated SNVs between populations are similar to those observed for random SNVs [12,15,17,49]. Individually, however, several disease-associated alleles appear to have increased in frequency due to positive selection in certain human populations, and thus may be responsible for large differences in disease prevalence between populations. Moreover, alleles that cause disease in contemporary environments may have been positively selected in ancestral environments. Positive selection results in large allele frequency differences between populations [6,9,50,51].

Genetic variants related to osteoporosis

Notably, the findings reported here show high variant frequency considered as a risk for genes related to metabolism and estrogen response, particularly in ancestral populations. The same is true for the *IL6* variant, which has a prevalence of 100%. This may be due to a selective advantage conferring a greater probability of survival to individuals hyper-reactive to aggressive stimuli that could represent a possible risk factor today, or due to a founding event. In addition, although our study does not include IL6 levels that would confirm this hypothesis, existing studies conducted in the Mexican population have found higher levels of IL6 associated with autoimmune conditions such as arthritis and other variants under study, which helps to support this hypothesis [29,52,53].

Conversely, the risk variants observed at very low frequencies were rs1544410, rs731236, rs7975232 (*VDR*), rs1801197 (*CALCR*), rs2073617 (*TNFRSF11B*), and rs1800247 (*BGLAP*), and were practically absent for rs1800012 (*COL1A1*), the latter two being highly differentiated from populations in the rest of the world (Figure 1). Moreover, the latter was previously identified in another study in a native Mexican population [47].

Analyzing each variant separately is important in each subpopulation, as it shows which risk variants are more prevalent in different population groups. In our sample, the analyzed variants were found to behave similarly across groups, and our results revealed several combinations with significant frequencies. One of the generated profiles comprises the risk alleles of the variants rs9340799, rs700518, rs1800795, and rs5030792. This profile was found in both Mestizo individuals and in the indigenous groups Huichol (16.7%), Maya (15%), Mazateca (9%), Nahua (8%), Purépecha (15.3%), Tarahumara (7%), and Mestizo (6%) (Table 4).

These results show that, in the Mazatec and Nahua groups, the risk allele of the rs1061624 variant, which has also been associated with a greater inflammatory response [54,55], contributed to the aforementioned profile. The Mestizo and Purépecha groups exhibited the same profile (Table 4), which could generate genetic interaction, further increasing the inflammatory response, in addition to the risk variants already mentioned.

The genetic architecture of bone metabolism regulation comprises many SNVs [56], which represent susceptibility factors for diseases such as osteoporosis in the general population. The variations analyzed in this study have been associated with low bone mass and increased bone fragility, making individuals more prone to fractures [57]. GWASs have detected different biomarkers and demonstrated their relevance in various populations, particularly European populations. The identification of different combinations in the population analyzed in this study can provide information on susceptibility profiles for low bone density, indicating that this population is the general population.

One of the profiles identified in this study could make individuals susceptible to various conditions besides osteoporosis, since the variants it comprises have been associated with various diseases: rs9340799 with breast cancer, osteoporosis, and osteoarthritis; rs700518 with hypertension, breast cancer, and osteoporosis; rs1800795 with cardiovascular disease, lupus erythematosus, and osteoporosis; and rs5030792 with inflammatory diseases and hypertension. Although the sample size per group is small, the variant was found in approximately 10% of all groups, indicating a risk variant frequency that should be taken into account in future studies [58,59].

However, this profile could have a greater impact on bone alterations due to the combined effect of these variant genes. The rs9340799 gene produces fewer receptors, resulting in a reduced response to estrogen. The rs700518 gene, which encodes the aromatase enzyme, decreases the protein’s functionality, thus reducing circulating estrogen levels, which can lead to reduced bone formation. The rs1800795 gene generates higher serum levels of IL6, which increases inflammatory activity, as does the rs5030792 gene, which is associated with intensified inflammatory responses. These cytokines increase bone resorption, and combined with reduced bone formation, the lower bone density in these individuals could make them more susceptible to bone diseases [52,60,61].

The study of risk variants associated with disease should not be limited to patients in whom their frequency is expected to be higher due to disease progression and genetic predisposition. It is also important to study them in the general population in order to determine their frequency and whether this population may have a higher risk of developing the disease due to genetic factors [62].

Implications for precision medicine

The small number of variants analyzed in this study does not allow for the generation of broader genetic profiles, which could provide more information. However, some studies have shown clinical relevance of profiles with a lower number of SNVs. Some of these variants were analyzed in Mexican patients with osteoporosis, and a profile of four SNVs was found to be associated with the response to alendronate treatment [63]. These same variants were included in our study. Even with a limited number of SNVs, the profiles generated from them can provide clinically relevant information about the genetic makeup of the Mexican population. Individuals with the profile analyzed above, in combination with other factors (including environmental), may be more susceptible to diseases associated with bone metabolism or involving high inflammatory responses, such as rheumatoid arthritis. Although it is an autoimmune disease, studies have found a potential causal association between genetically predicted RA and osteoporosis. Therefore, determining these profiles could be beneficial for both conditions [64].

A recent study in a mixed-race population of Mexico demonstrated the risk of osteoporosis and fractures associated with markers from both the estrogenic (particularly *ESR1* and *CYP19A1*) and the inflammatory pathways (*IL6* and *RANK/RANKL*). They demonstrated an additive or interactive effect between different genetic variations and gene–gene interactions, primarily within the same physiological pathway, and found that gene interaction with only two SNVs increases the risk of osteoporosis, with an OR = 2.1 [65].

Knowledge of the frequencies of risk variants and their combinations in the studied population could be advantageous in preventive medicine, as it could inform the development of measures to identify people at higher risk of developing a disease, potentially enabling its prevention. Studies assume that there are more Caucasian genetic backgrounds in admixed than native populations, as shown for these allele frequencies, although no difference was observed in this study (Table 4) [44,66]. Different habits and customs in terms of diet and physical activity among natives and Mestizos increase the differences between populations, but these populations carry the same habit-associated risks as urban Mestizos, so they could be considered to be at higher risk.

Allele profile analysis could lead to the development of new medical tools for diagnosing and managing individuals or populations with a high frequency of genetic risk factors, thus enabling the adoption of preventive measures and a reduction in healthcare costs. Larger and more in-depth studies that include phenotypic variables and populations with disease could elucidate the influence of these markers and their role in cardiovascular and inflammatory characteristics, as well as bone mineral density, in the Mexican population.

Regarding the current demographic transition in Mexican Mestizo populations, a steady increase in the elderly population has been observed. This increases the risk of various degenerative diseases such as cancer, chronic inflammatory bowel disease, other inflammatory diseases such as rheumatoid arthritis and multiple sclerosis, and metabolic diseases such as diabetes, hypertension, and the aforementioned bone disorders, such as osteoporosis [52,54,55]. According to demographic projections, in 20 years, one-third of the Mexican population will be over 50 years old. Knowledge of the frequency of alleles associated with a higher risk of degenerative traits and the combination of these factors could provide a basis for understanding the biological factors that contribute to these conditions in the tested population [47,67].

It is important to highlight this study’s limitations. There is a small sample size in each sub-group and a lack of additional population groups, which limits the applicability of the results to all populations. There is also a greater number of risk factors. Although participants declared themselves healthy, we lack information on disease phenotypes and the environmental factors to which they are exposed, which could provide further insights. However, the study’s main objective is to analyze intra-population variability and perform extra-population comparisons to obtain a more complete sample representative of the genetic structure of the Mexican population, and one of its main contributions is the inclusion of diverse population groups. This makes our sample more representative of the genetic structure of the Mexican population than if we had simply analyzed Mestizo individuals, and it could allow us to extrapolate the results not only to Mestizo and mixed-ethnic populations but also to other groups at higher risk of developing certain diseases.

Emphasizing that one of the main limitations is the lack of clinical and biochemical characteristics of the participants, and therefore the absence of genotype-phenotype association analysis, which, while not the objective of this study, could provide important information. Therefore, the potential effect of these variants in populations, especially native populations, requires further and in-depth analysis in subsequent studies. However, the high frequencies observed in these possible risk variants warrant further association studies in native populations.

This study represents only a few reports of biomedical markers of allelic risk in American natives that, compared with the variability in the Mestizo population, make it more difficult to address complex diseases. This is particularly relevant to understanding highly genetically stratified populations such as the Mexican population.

## 5. Conclusions

The detection of disease-associated gene variants in stratified populations, despite their small effect on phenotype, provides information about the genetic structure of each population. Characterization of allelic profiles can provide biomedical information to identify individuals at risk of developing bone and cartilage diseases, such as osteoporosis or arthritis, and to inform decisions about preventive interventions.

## Figures and Tables

**Table 1:** Description of genetic variants.

Gene	Protein	dbSNV	Consequence	Alleles	Biological Pathways	Association Studies/GWAS
*COL1A1*	Colagen1 α 1	rs1800012	Intron Variant	G>T	Structural	[20,21]
*ESR1*	Estrogen α Receptor	rs9340799	Intron Variant	A>G	Bone formation	[21,22,23,24]
*VDR*	Vitamin D Receptor	rs7975232	Intron Variant	C>A	Bone formation	[25]
*VDR*	Vitamin D Receptor	rs1544410	Intron Variant	C>A	Bone formation	[25,26,27]
*VDR*	Vitamin D Receptor	rs731236	Missense Variant	A>C	Bone formation	[25]
*CYP19A1*	Aromatase	rs700518	Amino acid change	T>A	Bone formation	[25]
*BGLAP*	Osteocalcin	rs1800247	Intron Variant	T>C	Structural	[28]
*CALCR*	Calcitonin Receptor	rs1801197	Missense Variant	A>T	Bone formation	[28]
*TGFB1*	Transforming Growth Factor-β1	rs1800469	Upstream Variant	A>T	Bone formation	[28]
*IL6*	Interleukin 6	rs1800795	Intron Variant	C>G	Bone resorption	[29]
*TNFRSF11B*	Osteoprotegerin	rs2073617	Upstream Variant	G>T	Bone formation	[30]
*PTHRI*	Type-1 Parathyroid Hormone Receptor	rs724449	Intron Variant	C>A	Bone resorption	[28]
*TNFRSF1B*	Tumour Necrosis Factor Receptor	rs1061624	3 Prime UTR Variant	A>G	Bone resorption	[31]
*TNFRSF1B*	Tumour Necrosis Factor Receptor	rs5030792	3 Prime UTR Variant	T>G	Bone resorption	[31]
*TNFRSF1B*	Tumour Necrosis Factor Receptor	rs3397	3 Prime UTR Variant	C>T	Bone resorption	[31]

**Table 2:** Demographic characteristics.

Population	Volunteers, *n*	Female Sex, *n* (%)	Male Sex, *n* (%)	City Council	Age (Years), Mean ± SD
Native American	304				
Huicholes	30	50	50	Mezquitic, Jalisco	34.8 ± 17.9
Mayas	39	73	27	Felipe Carrillo Puerto, Chemuyil, Quintana Roo	37.4 ± 16.5
Lacandones	30	60	40	Ocosingo, Chiapas	43.0 ± 16.0
Tzeltales	20	100	0	Tenejapa, Chiapas	38.9 ± 10.6
Mazatecas	35	64	36	San Miguel Soyaltepec, Oaxaca	45.8 ± 15.6
Nahuas	50	74	26	Tuxpan, Jalisco	46.0 ± 17.1
Purephechas	50	76	24	Zipiajo, Michoacán	49.9 ± 20.4
Tarahumaras	50	68	32	Carichi, Guazapares, Gpe y Calvo, Bocoina, Voigue, Chihuahua	44.4 ± 14.8
Mestizos	130				
Aguascalientes	32	68	32	Aguascalientes	32.9 ± 11.2
Jalisco	38	64	36	Ocotlán	28.4 ± 10.6
Yucatán	20	100	0	Mérida	35.8 ± 14.7
Chiapas	20	100	0	San Cristóbal de las Casas	39.6 ± 16.6
Chihuahua	20	40	60	Chihuahua	41.2 ± 18.6

**Table 3:** Allele frequencies for 15 SNVs associated with diseases. Sample size is shown below the population name.

Gene	dbSNV	Associated Allele	Huicholes (30)	Mayas (40)	Lacandones (30)	Tzeltales (20)	Mazatecas (34)	Nahuas (50)	Purépechas (50)	Tarahumaras (50)	Mestizos (130)
*COL1A1*	rs1800012	T	0	0.012	0	0	0	0	0	0.021	0.091
*ESR1*	rs9340799	A	0.833	0.759	0.650	0.750	0.875	0.844	0.927	0.813	0.687
*VDR*	rs7975232	A	0.383	0.333	0.325	0.397	0.379	0.310	0.217	0.276	0.362
*VDR*	rs1544410	A	0.042	0.050	0	0.125	0.093	0.068	0.067	0.184	0.307
*VDR*	rs731236	C	0.036	0.055	0.018	0.010	0.050	0.128	0.033	0.039	0.214
*CYP19A1*	rs700518	A	1.000	0.957	0.981	0.850	1.000	1.000	1.000	0.958	1.000
*BGLAP*	rs1800247	C	0.214	0.011	0.071	0.025	0.348	0.352	0.098	0.443	0.133
*CALCR*	rs1801197	T	0.333	0.222	0.017	0.300	0.212	0.430	0.422	0.532	0.417
*TGFB1*	rs1800469	T	0.250	0.324	0.448	0.300	0.383	0.082	0.061	0.104	0.280
*IL6*	rs1800795	G	1.000	1.000	1.000	1.000	1.000	1.000	1.000	1.000	1.000
*TNFRSF11B*	rs2073617	T	0.383	0.163	0.155	0.200	0.234	0.300	0.189	0.153	0.335
*PTHRI*	rs724449	A	0.617	0.455	0.350	0.400	0.567	0.514	0.625	0.625	0.564
*TNFRSF1B*	rs1061624	A	0.367	0.343	0.317	0.300	0.453	0.398	0.398	0.427	0.393
*TNFRSF1B*	rs5030792	T	0.833	0.785	0.833	1.000	1.000	0.708	0.977	0.837	0.939
*TNFRSF1B*	rs3397	C	0.100	0.244	0.083	0.350	0.061	0.210	0.256	0.330	0.302

**Table 4:** Allelic profile frequencies. The alleles in red are considered risky. The most frequent combinations among the 15 markers per population are shown.

Gene	*DbSNV*	*Huicholes*	*Mayas*	*Mazatecas*	*Nahuas*	*Purépechas*			*Tarahumaras*		*Mestizos*
*VDR*	rs1544410	G	G	G	G	G	G	G	G	G	G
*VDR*	rs7975232	C	C	C	C	C	C	A	C	C	A
*VDR*	rs731236	T	T	T	T	T	T	T	T	T	T
*COL1A1*	rs1800012	G	G	G	G	G	G	G	G	G	G
*ESR1*	rs9340799	A	A	A	A	A	A	A	A	A	A
*CYP19A1*	rs700518	A	A	A	A	A	A	A	A	A	A
*BGLAP*	rs1800247	T	T	T	T	T	T	T	T	T	T
*CALCR*	rs1801197	C	C	C	C	C	T	T	T	C	C
*TGFB1*	1800469	C	C	C	C	C	C	C	C	C	T
*IL6*	rs1800795	G	G	G	G	G	G	G	G	G	G
*PTHR1*	rs724449	A	G	A	A	A	A	G	A	G	A
*TNFRSF11B*	rs2073617	C	C	C	C	C	C	C	C	C	C
*TNFRSF1B*	rs1061624	G	G	A	A	G	A	G	G	G	A
*TNFRSF1B*	rs5030792	T	T	T	T	T	T	T	T	T	T
*TNFRSF1B*	rs3397	T	T	T	T	T	T	T	T	T	T
*Frequencies (%)*		16.7	15.1	9.2	8	15.3	6.2	6.1	7	6	6

**Table 5:** Population pairwise *Fst*. Distance measurement method: pairwise differences. *Fst* distances (downward diagonal); *p* values (upward diagonal).

	Mazatecas	Purepechas	Tarahumaras	Nahuas	Mayas	Tzeltales	Lacandones	Huicholes	Mestizos	European	African	Mexicoamerican	Asian
Mazatecas	0.00000	0.00000	0.00000	0.00000	0.00178	0.00089	0.04049	0.03663	0.00000	0.00000	0.00000	0.00010	0.00000
Purepechas	0.06754	0.00000	0.11375	0.29631	0.06395	0.12929	0.00000	0.00000	0.00000	0.00000	0.00000	0.00000	0.00000
Tarahumaras	0.05885	0.00341	0.00000	0.07494	0.59054	0.48490	0.00000	0.00020	0.00000	0.00000	0.00000	0.00000	0.00000
Nahuas	0.03941	−0.00092	0.00649	0.00000	0.03406	0.10474	0.00000	0.15068	0.00000	0.00000	0.00000	0.00000	0.00000
Mayas	0.02824	0.00639	−0.00427	0.01083	0.00000	0.87575	0.00040	0.00158	0.00446	0.00000	0.00000	0.00000	0.00000
Tzeltales	0.04252	0.00461	−0.00390	0.00812	−0.01302	0.00000	0.01020	0.01089	0.06465	0.00000	0.00000	0.00010	0.00000
Lacandones	0.01145	0.08574	0.06204	0.04984	0.03300	0.03025	0.00000	0.00198	0.00020	0.00000	0.00000	0.00000	0.00000
Huicholes	0.01051	0.03326	0.03868	0.00357	0.02710	0.02547	0.02542	0.00000	0.00366	0.00000	0.00000	0.00030	0.00000
Mestizos	0.03145	0.04301	0.02297	0.02124	0.01319	0.00849	0.02628	0.01611	0.00000	0.00000	0.00000	0.00010	0.00000
European	0.08384	0.09895	0.08796	0.08450	0.07011	0.06522	0.09109	0.07249	0.04369	0.00000	0.00000	0.00000	0.00000
African	0.27738	0.25112	0.23284	0.23861	0.23235	0.22110	0.29991	0.23754	0.17785	0.10007	0.00000	0.00000	0.00000
Mexicoamerican	0.03174	0.09119	0.06893	0.06552	0.04662	0.05413	0.04467	0.03618	0.02298	0.03626	0.17588	0.00000	0.00000
Asian	0.18682	0.22266	0.18361	0.19343	0.17546	0.17238	0.20161	0.16414	0.12056	0.14962	0.15063	0.09277	0.00000

## Data Availability

The original contributions presented in this study are included in the article/Supplementary Materials. Further inquiries can be directed to the corresponding author.

## Supplementary Materials

The following supporting information can be downloaded at https://www.mdpi.com/article/10.3390/biomedicines14071470/s1: Table S1: Oligonucleotide primers, Table S2–S16: Genotypic frequencies and Hardy Weinberg equilibrium, Table S17–S49 Fst and *p* test results by variation and between variants in all populations, Table S50–S58: linkage disequilibrium analysis by variation and between variants in all populations, Figure S1: linkage disequilibrium map and block.

## References

[1] Visscher P.M., Wray N.R., Zhang Q., Sklar P., McCarthy M.I., Brown M.A., Yang J. (2017). 10 Years of GWAS Discovery: Biology, Function, and Translation. Am. J. Hum. Genet..

[2] Dehghan A. (2018). Genome-Wide Association Studies. Methods Mol. Biol..

[3] Sabik O.L., Farber C.R. (2017). Using GWAS to identify novel therapeutic targets for osteoporosis. Transl. Res..

[4] Tam V., Patel N., Turcotte M., Bossé Y., Paré G., Meyre D. (2019). Benefits and limitations of genome-wide association studies. Nat. Rev. Genet..

[5] Padmanabhan S., Dominiczak A.F. (2021). Genomics of hypertension: The road to precision medicine. Nat. Rev. Cardiol..

[6] International HapMap Consortium (2003). The International HapMap Project. Nature.

[7] Redman M.G., Horton R.H., Carley H., Lucassen A. (2024). Ancestry, race and ethnicity: The role and relevance of language in clinical genetics practice. J. Med. Genet..

[8] Lohmueller K.E., Mauney M.M., Reich D., Braverman J.M. (2006). Variants associated with common disease are not unusually differentiated in frequency across populations. Am. J. Hum. Genet..

[9] Myles S., Davison D., Barrett J., Stoneking M., Timpson N. (2008). Worldwide population differentiation at disease-associated SNPs. BMC Med. Genom..

[10] Manolio T.A., Collins F.S., Cox N.J., Goldstein D.B., Hindorff L.A., Hunter D.J., McCarthy M.I., Ramos E.M., Cardon L.R., Chakravarti A. (2009). Finding the missing heritability of complex diseases. Nature.

[11] Liu Y.J., Zhang L., Papasian C.J., Deng H.W. (2014). Genome-wide Association Studies for Osteoporosis: A 2013 Update. J. Bone Metab..

[12] Freedman M.L., Reich D., Penney K.L., McDonald G.J., Mignault A.A., Patterson N., Gabriel S.B., Topol E.J., Smoller J.W., Pato C.N. (2004). Assessing the impact of population stratification on genetic association studies. Nat. Genet..

[13] Moreno-Estrada A., Gignoux C.R., Fernández-López J.C., Zakharia F., Sikora M., Contreras A.V., Acuña-Alonzo V., Sandoval K., Eng C., Romero-Hidalgo S. (2014). Human genetics. The genetics of Mexico recapitulates Native American substructure and affects biomedical traits. Science.

[14] Sun S., Dong B., Zou Q. (2021). Revisiting genome-wide association studies from statistical modelling to machine learning. Brief. Bioinform..

[15] Marchini J., Cardon L.R., Phillips M.S., Donnelly P. (2004). The effects of human population structure on large genetic association studies. Nat. Genet..

[16] Okser S., Pahikkala T., Aittokallio T. (2013). Genetic variants and their interactions in disease risk prediction, machine learning and network perspectives. BioData Min..

[17] Cross D.S., Ivacic L.C., Stefanski E.L., McCarty C.A. (2010). Population based allele frequencies of disease associated polymorphisms in the Personalized Medicine Research Project. BMC Genet..

[18] Smith J.L., Tcheandjieu C., Dikilitas O., Iyer K., Miyazawa K., Hilliard A., Lynch J., Rotter J.I., Chen Y.-D.I., Sheu W.H.-H. (2024). Multi-Ancestry Polygenic Risk Score for Coronary Heart Disease Based on an Ancestrally Diverse Genome-Wide Association Study and Population-Specific Optimization. Circ. Genom. Precis. Med..

[19] Lohmueller K.E., Pearce C.L., Pike M., Lander E.S., Hirschhorn J.N. (2003). Meta-analysis of genetic association studies supports a contribution of common variants to susceptibility to common disease. Nat. Genet..

[20] Falcón-Ramírez E., Hidalgo-Bravo A., Barredo-Prieto B.A., Pineda-Gómez E., Valdés-Flores M. (2016). Association of the COL1A1 gene polymorphisms in Mexican postmenopausal women with fracture or with low bone mineral density at the hip. Aging Clin. Exp. Res..

[21] Rojano-Mejía D., Coral-Vázquez R.M., Espinosa L.C., López-Medina G., Aguirre-García M.C., Coronel A., Canto P. (2013). JAG1 and COL1A1 polymorphisms and haplotypes in relation to bone mineral density variations in postmenopausal Mexican-Mestizo Women. Age.

[22] Borgonio-Cuadra V.M., González-Huerta C., Duarte-Salazár C., de Los Ángeles Soria-Bastida M., Cortés-González S., Miranda-Duarte A. (2012). Analysis of estrogen receptor alpha gene haplotype in Mexican mestizo patients with primary osteoarthritis of the knee. Rheumatol. Int..

[23] Wang K.J., Shi D.Q., Sun L.S., Jiang X., Lü Y.Y., Dai J., Chen D.Y., Xu Z.H., Jiang Q. (2012). Association of estrogen receptor alpha gene polymorphisms with bone mineral density: A meta-analysis. Chin. Med. J..

[24] Ren Y., Tan B., Yan P., You Y., Wu Y., Wang Y. (2015). Association between polymorphisms in the estrogen receptor alpha gene and osteoarthritis susceptibility: A meta-analysis. BMC Musculoskelet. Disord..

[25] González-Mercado A., Sánchez-López J., Regla-Nava J., Gámez-Nava J., González-López L., Duran-Gonzalez J., Celis A., Perea-Díaz F., Salazar-Páramo M., Ibarra B. (2013). Association analysis of vitamin D receptor gene polymorphisms and bone mineral density in postmenopausal Mexican-Mestizo women. Genet. Mol. Res..

[26] McClure L., Eccleshall T.R., Gross C., Villa M.L., Lin N., Ramaswamy V., Kohlmeier L., Kelsey J.L., Marcus R., Feldman D. (1997). Vitamin D receptor polymorphisms, bone mineral density, and bone metabolism in postmenopausal Mexican-American women. J. Bone Miner. Res..

[27] Avila M., Prado C., Ventura M.-D., Mora C., Briones D., Valdez H., Hurtado M.E., Lindholm B., Qureshi A., Castillo-Henkel C. (2010). Vitamin D receptor gene, biochemical bone markers and bone mineral density in Mexican women on dialysis. Nephrol. Dial. Transplant..

[28] Xu X.H., Dong S.S., Guo Y., Yang T.L., Lei S.F., Papasian C.J., Zhao M., Deng H.W. (2010). Molecular genetic studies of gene identification for osteoporosis: The 2009 update. Endocr. Rev..

[29] Magaña J.J., Gómez R., Cisneros B., Casas L., Valdés-Flores M. (2008). Association of interleukin-6 gene polymorphisms with bone mineral density in Mexican women. Arch. Med. Res..

[30] Rojano-Mejía D., Coral-Vázquez R.M., Espinosa L.C., Romero-Hidalgo S., López-Medina G., García M.d.C.A., Coronel A., Ibarra R., Canto P. (2012). TNFRSF11B gene haplotype and its association with bone mineral density variations in postmenopausal Mexican-Mestizo women. Maturitas.

[31] Chen W., Xu H., Wang X., Gu J., Xiong H., Shi Y. (2015). The tumor necrosis factor receptor superfamily member 1B polymorphisms predict response to anti-TNF therapy in patients with autoimmune disease: A meta-analysis. Int. Immunopharmacol..

[32] Bender K. (2005). SNaPshot for pharmacogenetics by minisequencing. Methods Mol. Biol..

[33] Excoffier L., Lischer H.E. (2010). Arlequin suite ver 3.5: A new series of programs to perform population genetics analyses under Linux and Windows. Mol. Ecol. Resour..

[34] Tereba A. (2001). Tools for Analysis of Population Statistics. Profiles in DNA, Promega Corporation. https://www.promega.com.

[35] Pritchard J.K., Stephens M., Donnelly P. (2000). Inference of population structure using multilocus genotype data. Genetics.

[36] Falush D., Stephens M., Pritchard J.K. (2003). Inference of population structure using multilocus genotype data: Linked loci and correlated allele frequencies. Genetics.

[37] Falush D., Stephens M., Pritchard J.K. (2007). Inference of population structure using multilocus genotype data: Dominant markers and null alleles. Mol. Ecol. Notes.

[38] Chen T., Zhang H., Mazumder R., Lin X. (2024). SPLENDID incorporates continuous genetic ancestry in biobank-scale data to improve polygenic risk prediction across diverse populations. bioRxiv.

[39] Lander E., Schork N. (1994). Genetic Dissection of Complex Traits. Science.

[40] Chacón-Duque J.-C., Adhikari K., Fuentes-Guajardo M., Mendoza-Revilla J., Acuña-Alonzo V., Barquera R., Quinto-Sánchez M., Gómez-Valdés J., Martínez P.E., Villamil-Ramírez H. (2018). Latin Americans show wide-spread Converso ancestry and imprint of local Native ancestry on physical appearance. Nat. Commun..

[41] Rubí-Castellanos R., GarcíaAceves M.E., Aguilar-Veláquez J.A., Gabriela MartínezCortés G., Nuño-Arana I., Rangel-Villalobos H. (2021). Análisis filogenético vía paterna de nueve etnias de México. Estudios de Antropología Biológica.

[42] Mariscal-Ramos C., Cortes-Trujllo I., Martínez-Cortés G., Arana I.N., Rangel-Villalobos H. (2024). Population expansion, larger, and more homogeneous native American ancestry among Mexican mestizo populations based on 10 X-chromosome STR loci (X-STR decaplex system). Am. J. Hum. Biol..

[43] Rangel-Villalobos H., Martínez-Sevilla V.M., Martínez-Cortés G., Aguilar-Velázquez J.A., Sosa-Macías M., Rubi-Castellanos R., González-Martín A. (2016). Importance of the geographic barriers to promote gene drift and avoid pre- and post-Columbian gene flow in Mexican native groups: Evidence from forensic STR Loci. Am. J. Phys. Anthropol..

[44] Martínez-Cortés G., Zúñiga-Castellanos R.A., García-Aceves M.E., Salcido V.H., Cortés-Trujillo I., Rangel-Villalobos H. (2017). Genetic diversity of Mexican-Mestizo populations using 114 INDEL polymorphisms. Forensic Sci. Int. Genet. Suppl. Ser..

[45] García-Ortiz H., Barajas-Olmos F., Contreras-Cubas C., Cid-Soto M.Á., Córdova E.J., Centeno-Cruz F., Mendoza-Caamal E., Cicerón-Arellano I., Flores-Huacuja M., Baca P. (2021). The genomic landscape of Mexican Indigenous populations brings insights into the peopling of the Americas. Nat. Commun..

[46] García-Ortiz H., Barajas-Olmos F., Contreras-Cubas C., Reynolds A.W., Flores-Huacuja M., Snow M., Ramos-Madrigal J., Mendoza-Caamal E., Baca P., López-Escobar T.A. (2022). Unraveling Signatures of Local Adaptation among Indigenous Groups from Mexico. Genes.

[47] Nuño-Arana I., Sahagún-Núñez V.D.R., Muñoz-Valle J.F., Sandoval L., Pinto-Escalante D., Páez-Riberos L.A., Lazalde B., Maldonado-González M., Rangel-Villalobos H. (2012). Distribution of three SNPs related to low bone mineral density in Amerindian groups and Mestizos from Mexico. Am. J. Hum. Biol..

[48] Porras-Hurtado L., Ruiz Y., Santos C., Phillips C., Carracedo A., Lareu M.V. (2013). An overview of STRUCTURE: Applications, parameter settings, and supporting software. Front. Genet..

[49] Kiel D.P., Demissie S., Dupuis J., Lunetta K.L., Murabito J.M., Karasik D. (2007). Genome-wide association with bone mass and geometry in the Framingham Heart Study. BMC Med. Genet..

[50] Dvornyk V., Long J.-R., Xiong D.-H., Liu P.-Y., Zhao L.-J., Shen H., Zhang Y.-Y., Liu Y.-J., Rocha-Sanchez S., Xiao P. (2004). Current limitations of SNP data from the public domain for studies of complex disorders: A test for ten candidate genes for obesity and osteoporosis. BMC Genet..

[51] Myles S., Hradetzky E., Engelken J., Lao O., Nürnberg P., Trent R.J., Wang X., Kayser M., Stoneking M. (2007). Identification of a candidate genetic variant for the high prevalence of type II diabetes in Polynesians. Eur. J. Hum. Genet..

[52] Posadas-Sánchez R., López-Uribe Á.R., Fragoso J.M., Vargas-Alarcón G. (2024). Interleukin 6 polymorphisms are associated with cardiovascular risk factors in premature coronary artery disease patients and healthy controls of the GEA Mexican study. Exp. Mol. Pathol..

[53] Miranda-Duarte A., de León-Suárez V.P., Hidalgo-Bravo A., Velázquez-Cruz R., Ramírez-Pérez E., Martínez-Ramírez O.C., Castro-Hernández C., Barredo-Prieto B., Casas-Avila L. (2026). Gene-gene and gene-environment interactions of CYP19A1, ESR1, IL6, IL6R, IL1β, RANK, and RANKL variants in relation to osteoporosis and hip fracture risk in Mexican women. Front. Aging.

[54] Pérez-Hernández N., Posadas-Sánchez R., Vargas-Alarcón G., Cazarín-Santos B.G., Miranda-Duarte A., Rodríguez-Pérez J.M. (2020). Genetic Variants and Haplotypes in OPG Gene Are Associated with Premature Coronary Artery Disease and Traditional Cardiovascular Risk Factors in Mexican Population: The GEA Study. DNA Cell Biol..

[55] Katsanis N., Mourtzi N., Quinto-Cortés C.D., Martagon A.J., Ioannidis A.G., De La Vega F.M., Gulcher J., Lee M.T.M., Faghihi M.A., Lopez-Pineda A. (2025). Analysis of a deeply-phenotyped familial hypercholesterolemia cohort from Mexico shows a role for both rare and common alleles across known dyslipidemia genes and reveals structural variation in a novel locus. Hum. Genom..

[56] Malavašič P., Lojk J., Lovšin M.N., Marc J. (2025). Recent Advances in Experimental Functional Characterization of GWAS Candidate Genes in Osteoporosis. Int. J. Mol. Sci..

[57] Tobias J.H., Karasik D. (2021). Editorial: Recent Advances in the Genetics of Osteoporosis. Front. Endocrinol..

[58] Carrillo-Moreno D.I., Eduardo Figuera L., Zuniga González G.M., Puebla Perez A.M., Jesus Moran Mendoza A., Gallegos Arreola M.P. (2019). Association of rs2234693 and rs9340799 polymorphisms of ESR1 gene in breast cancer of Mexican population. J. BUON.

[59] Richards J.B., Zheng H.F., Spector T.D. (2012). Genetics of osteoporosis from genome-wide association studies: Advances and challenges. Nat. Rev. Genet..

[60] Riancho J.A., Sañudo C., Valero C., Pipaón C., Olmos J.M., Mijares V., Fernández-Luna J.L., Zarrabeitia M.T. (2009). Association of the aromatase gene alleles with BMD: Epidemiological and functional evidence. J. Bone Miner. Res..

[61] Boone S.D., Baumgartner K.B., Baumgartner R.N., Connor A.E., Pinkston C.M., Rai S.N., Riley E.C., Hines L.M., Giuliano A.R., John E.M. (2014). Associations between CYP19A1 polymorphisms, Native American ancestry, and breast cancer risk and mortality: The Breast Cancer Health Disparities Study. Cancer Causes Control.

[62] Wu Q., Dai J., Liu J., Wu L. (2025). Bridging Genomic Research Disparities in Osteoporosis GWAS: Insights for Diverse Populations. Curr. Osteoporos. Rep..

[63] Villagómez Vega A., Gámez Nava J.I., Ruiz González F., Pérez Romero M., Trujillo Rangel W.Á., Nuño Arana I. (2023). Influence of the Osteogenomic Profile in Response to Alendronate Therapy in Postmenopausal Women with Osteoporosis: A Retrospective Cohort Study. Genes.

[64] Deng Y., Wong M.C.S. (2023). Association Between Rheumatoid Arthritis and Osteoporosis in Japanese Populations: A Mendelian Randomization Study. Arthritis Rheumatol..

[65] Rottura M., Pirrotta I., Giorgi D.A., Irrera N., Arcoraci V., Mannino F., Campisi R., Bivacqua C., Patanè L., Costantino G. (2025). Genetic Polymorphisms on TNFA, TNFRSF1A, and TNFRSF1B Genes Predict the Effectiveness of Anti-TNF-α Treatment in Inflammatory Bowel Disease Patients. Biomedicines.

[66] Manolagas S.C., Jilka R.L. (1995). Bone marrow, cytokines, and bone remodeling. Emerging insights into the pathophysiology of osteoporosis. N. Engl. J. Med..

[67] CONAPO (2014). Consejo Nacional de Población y Vivienda. https://www.gob.mx/conapo/documentos/la-situacion-demografica-de-mexico-2014.

