# Supplementary material for: Genetic Diversity Analysis of Risk Variants Associated with Bone and Cartilage Metabolism in Nine Mexican Subpopulations
Source: Biomedicines. 2026 Jun 29;14(7):1470. doi: 10.3390/biomedicines14071470 (PMC13405977; doi:10.3390/biomedicines14071470)
Supplement: Supplementary file 1 [file biomedicines-14-01470-s001.zip › biomedicines-4299598-supplementary.pdf]

Supplementary Material for

Genetic Diversity Analysis of Risk Variants Associated with Bone and Cartilage Metabolism in Nine Mexican Subpopulations

Table S1. Oligonucleotide Primers

**Gene/dbSNV**	**Oligonucleotide Primer**
COL1A1 rs1800012	TTTTTTTTTTTTTTTTTGCCCCCGCCACCCCACCTGCCCAGGGAATG
ESR1 rs9340799	GAGACCCTGAGTGTGGTCT
VDR rs7975232	TTTTTTTTTTTTTTTTTTTAGAAGAAGGCACAGGAGCTCTCAGCTGGGC
VDR rs1544410	GCAGAGCCTGAGTATTGGGAATG
VDR rs731236	TTTTTTTGGGTGCAGGACGCCGCGCTGAT
CYP19A1 rs700518	TTTTTTTTTTTTTTGTGCCTGCAACTACTACAACCGGGT
BGLAP rs1800247	TTTTTTTTCCCAGCAGCCGCAGCTCCCAACCACAATATCCT
CALCR rs1801197	TTTTTTTTCGCCTTGGTTGTTGGCTGGTTCATTCCTC
TGFB1 rs1800469	TGCCTCCTGACCCTTCCATCC
IL6 rs1800795	CTAGTTGTGTCTTGC
OPG rs2073617	TTTTCCTTTCCGCCCCAGCCCTGAAAGCGTTAA
PTHR1 rs724449	ATTGGATTTTTAATGTTGACTTTATCATTTC
TNFRSF1B	TTTTTTTTTTTTTCTGACCTGCAGGCCAAGAGCAGAGGCAGCG

rs1061624	
TNFRSF1B rs5030792	TTTTTTTTTTTTTTTTTTTTTTCTGCAGGCCAAGAGCAGAGGCAGCGAGTTG
TNFRSF1B rs3397	TTTTTTTTTTTTTTTCATGCCCAGCCAGCCTTCCGAGAGGGACAC

Genotypic frequencies and Hardy-Weinberg equilibrium

Table S2. *COL1A1* (rs1800012)

	Genotype n (%)			EHW
**rs1800012**	**G/G**	**G/T**	**T/T**	*p-value*
**Mestizos**	106 (83.5)	19 (14.9)	1 (1.6)	0.156
**Huicholes**	30 (100)	0	0	N/A
**Mayas**	84 (97.7)	2 (2.3)	0	**0.001**
**Mazatecas**	32 (100)	0	0	N/A
**Nahuas**	48 (98)	1 (2)	0	1.000
**Purépechas**	48 (98)	1 (2)	0	1.000
**Tarahumaras**	46 (96)	2 (4)	0	1.000

* N/A, not applicable (monomorphic)

Table S3. *ESR1* (rs9340799)

rs9340799	Genotype n (%) A/A	A/G	G/G	EHW p-value
**Mestizos**	49 (39.8)	71 (57.8)	3 (2.4)	**0.000**
**Huicholes**	20 (66.6)	10 (33.3)	0 (0)	0.567
**Mayas**	52 (61.2)	25 (29.4)	8 (9.4)	0.075
**Mazatecas**	24 (75)	8 (25)	0	**0.015**
**Nahuas**	36 (75)	9 (18.7)	3 (6.3)	0.070
**Purépechas**	41 (85.4)	7 (14.6)	0	1.000
**Tarahumaras**	32 (66.6)	14 (29.2)	2 (4.2)	0.674

Table S4. *VDR* (rs7975232)

rs7975232	Genotype n (%) C/C	C/A	A/A	EHW p-value
**Mestizos**	49 (39.8)	59 (48)	15 (12.2)	**0.006**
**Huicholes**	8 (26.7)	21 (70)	1 (3.3)	1.000
**Mayas**	35 (40.2)	46 (52.8)	6 (6.9)	1.000
**Mazatecas**	10 (34.5)	16 (55.2)	3 (10.3)	0.187
**Nahuas**	21 (42)	26 (52)	3 (6)	0.133
**Purépechas**	29 (63)	14 (30.4)	3 (6.6)	1.000
**Tarahumaras**	27 (55.1)	17 (34.7)	5 (10.2)	**0.005**

Table S5. *VDR* (rs1544410)

rs1544410	Genotype n (%) G/G	G/A	A/A	EHW p-value
**Mestizos**	33 (7)	5 (10)	9 (20)	**0.001**
**Huicholes**	18 (90)	2 (10)	0	**0.019**
**Mayas**	72 (90)	8 (10)	0	0.447
**Mazatecas**	18 (90)	1 (5)	1 (5)	0.461

**Nahuas**	29 (87.9)	3 (9.1)	1 (3)	0.332
**Purépechas**	38 (97.4)	1 (2.6)	0	0.414
**Tarahumaras**	11 (29.7)	26 (70)	1 (0.3)	0.474

Table S6.*VDR* (rs731236)

	Genotype n (%)			EHW
**rs731236**	**T/T**	**T/C**	**C/C**	*p-value*
**Mestizos**	66 (63)	33 (31.4)	6 (5.7)	0.559
**Huicholes**	26 (92.9)	2 (7.1)	0	1.000
**Mayas**	73 (89)	9 (11)	0	1.000
**Mazatecas**	27 (95)	3 (5)	0	1.000
**Nahuas**	35 (81.4)	5 (11.6)	3 (7)	**0.011**
**Purépechas**	43 (93.5)	3 (6.5)	0	1.000
**Tarahumaras**	35 (92.2)	3 (7.9)	0	1.000

Table S7. *CYP19A1* (rs700518)

	Genotype n (%)			EHW
**rs700518**	**G/G**	**G/A**	**A/A**	*p-value*
**Mestizos**	0	0	103 (100)	N/A
**Huicholes**	0	0	30 (100)	N/A
**Mayas**	3 (3.7)	1 (1.2)	77 (95)	**0.000**
**Mazatecas**	0	0	24 (100)	N/A
**Nahuas**	0	0	46 (100)	N/A
**Purépechas**	0	0	46 (100)	N/A
**Tarahumaras**	0	5 (10.2)	44 (89.8)	1.000

* N/A, not applicable (monomorphic)

Table S8. ***BGLAP*** **(rs1800247)**

	Genotype n (%)			EHW
**rs1800247**	**C/C**	**C/T**	**T/T**	*p-value*
**Mestizos**	0	28 (26.7)	77 (73.3)	0.309
**Huicholes**	0	12 (42.9)	16 (57.1)	0.297
**Mayas**	3 (3.5)	13 (15.1)	70 (81.4)	0.077
**Mazatecas**	2 (9)	12 (52)	9 (39)	0.661
**Nahuas**	4 (9)	23 (52.3)	17 (38.6)	0.527
**Purépechas**	0	7 (17)	34 (83)	0.314
**Tarahumaras**	5 (14.3)	21 (60)	9 (25.7)	0.317

Table S9. ***CALCR*** **(rs1801197)**

	Genotype n (%)			EHW
**rs1801197**	**C/C**	**C/T**	**T/T**	*p-value*
**Mestizos**	42 (35)	56 (47)	22 (18)	0.707
**Huicholes**	15 (50)	10 (33.3)	5 (16.7)	0.218
**Mayas**	54 (61.3)	29 (33)	5 (5.7)	1.000
**Mazatecas**	22 (66.7)	8 (24.2)	3 (9)	0.121
**Nahuas**	17 (34)	23 (46)	10 (20)	0.772
**Purépechas**	13 (28.9)	28 (62.2)	4 (8.9)	0.127
**Tarahumaras**	9 (19.2)	26 (55.3)	12 (25.5)	0.239

Table S10. ***TGFBI*** **(rs1800469)**

	Genotype n (%)			EHW
**rs1800469**	**C/C**	**C/T**	**T/T**	*p-value*

**Mestizos**	58 (45.6)	67 (52.7)	2 (1.8)	**0.000**
**Huicholes**	15 (50)	15 (50)	0	0.143
**Mayas**	31 (36.5)	53 (62.4)	1 (1.1)	**0.001**
**Mazatecas**	10 (33.3)	17 (56.7)	3 (10)	0.265
**Nahuas**	41 (83.7)	8 (16.3)	0	1.000
**Purépechas**	44 (89.8)	4 (8.2)	1 (2)	0.131
**Tarahumaras**	38 (79.2)	10 (20.8)	0	1.000

Table S11. *IL6* (rs1800795)

	**Genotype n (%)**			**EHW**
**rs1800795**	**G/G**	**G/C**	**C/C**	***p-value***
**Mestizos**	122 (100)	0	0	N/A
**Huicholes**	30 (100)	0	0	N/A
**Mayas**	87 (100)	0	0	N/A
**Mazatecas**	29 (100)	0	0	N/A
**Nahuas**	47 (100)	0	0	N/A
**Purépechas**	46 (100)	0	0	N/A
**Tarahumaras**	49 (100)	0	0	N/A

* N/A, not applicable (monomorphic)

Table S12. *TNFRSF11B* (rs2073617)

	**Genotype n (%)**			**EHW**
**rs2073617**	**C/C**	**C/T**	**T/T**	***p-value***
**Mestizos**	57 (44.9)	55 (43.3)	15 (11.8)	0.841
**Huicholes**	11 (36.7)	15 (50)	4 (13.3)	1.000
**Mayas**	62 (72)	20 (23)	4 (5)	0.271
**Mazatecas**	18 (56)	13 (41)	1 (3)	0.652

**Nahuas**	23 (46)	24 (48)	3 (6)	0.496
**Purépechas**	26 (62.2)	19 (37.8)	0	0.314
**Tarahumaras**	34 (69.4)	15 (30.6)	0	0.585

Table S13. *PTHRI* (rs724449)

	Genotype n (%)			EHW
**rs724449**	**G/G**	**G/A**	**A/A**	*p-value*
**Mestizos**	24 (19.2)	61 (48.8)	40 (32)	1.000
**Huicholes**	5 (16.7)	13 (43.3)	12 (40)	0.702
**Mayas**	25 (28.4)	46 (52.3)	17 (19.3)	0.605
**Mazatecas**	7 (23)	12 (40)	11 (37)	0.346
**Nahuas**	9 (25)	19 (52.8)	8 (22.2)	1.000
**Purépechas**	6 (13.6)	21 (47.8)	17 (38.6)	1.000
**Tarahumaras**	3 (11.1)	13 (48.1)	11 (40.8)	1.000

Table S14. *TNFRSF1B* (rs1061624)

	Genotype n (%)			EHW
**rs1061624**	**A/A**	**A/G**	**G/G**	*p-value*
**Mestizos**	20 (15.9)	59 (46.8)	47 (37.3)	0.763
**Huicholes**	2 (6.7)	18 (60)	10 (33.3)	0.230
**Mayas**	14 (16.3)	31 (36)	41 (47.7)	0.103
**Mazatecas**	6 (19)	17 (53)	9 (28)	1.000
**Nahuas**	8 (16.3)	23 (47.9)	18 (36.7)	1.000
**Purépechas**	3 (6.1)	33(67.3)	13 (26.6)	**0.007**
**Tarahumaras**	9 (18.8)	22 (45.8)	17 (35.4)	1.000

Table S15. ***TNFRSF1B*** **(rs5030792)**

	Genotype n (%)			EHW
**rs5030792**	**T/T**	**T/G**	**G/G**	*p-value*
**Mestizos**	109 (89)	11 (9)	2 (2)	0.060
**Huicholes**	20 (66.7)	10 (33.3)	0	0.559
**Mayas**	56 (65.1)	23 (26.8)	7 (8.1)	0.060
**Mazatecas**	28 (100)	0	0	N/A
**Nahuas**	26 (54.2)	16 (33.3)	6 (12.5)	0.214
**Purépechas**	43 (95.6)	2 (4.4)	0	1.000
**Tarahumaras**	34 (79.1)	4 (9.3)	5 (11.6)	**0.001**

* N/A, not applicable (monomorphic)

Table S16. ***TNFRSF1B*** **(rs3397)**

	Genotype n (%)			EHW
**rs3397**	**T/T**	**T/C**	**C/C**	*p-value*
**Mestizos**	62 (49)	52 (41)	12 (10)	0.832
**Huicholes**	26 (86.6)	2 (6.7)	2 (6.7)	0.015
**Mayas**	51 (58)	31 (35)	6 (7)	0.763
**Mazatecas**	29 (88)	4 (12)	0	1.000
**Nahuas**	30 (60)	19 (38)	1 (2)	0.685
**Purépechas**	23 (51.1)	21 (46.7)	1 (2.2)	0.238
**Tarahumaras**	21 (44.7)	23 (48.9)	3 (6.4)	0.379

Fst and *p* test results by variation and between variants in all populations:

Table S17. rs7975232 (VDR) pairwise Fst

	MAZATECAS	PUREPECHAS	TARAHUMARAS	NAHUAS	MAYAS	TZELTALES	LACANDONES	HUICHOLES	MESTIZOS_JALISCO	EUROPEO	AFRICANO	MEXICOAMERICANOS	ASIATICOS
**MAZATECAS**	0.00000												
**PUREPECHAS**	0.02699	0.00000											
**TARAHUMARAS**	-0.00240	-0.00152	0.00000										
**NAHUAS**	-0.00911	0.00720	-0.00873	0.00000									
**MAYAS**	-0.00427	-0.00268	-0.01182	-0.01034	0.00000								
**TZELTALES**	-0.00017	-0.01518	-0.01623	-0.01158	-0.01765	0.00000							
**LACANDONES**	-0.01173	0.06225	0.01962	0.00717	0.01731	0.02638	0.00000						
**HUICHOLES**	-0.01065	0.06494	0.02168	0.00890	0.01934	0.02866	-0.01722	0.00000					
**MESTIZOS_JALISCO**	-0.01013	0.03927	0.00944	0.00141	0.00757	0.01290	-0.00814	-0.00730	0.00000				
**EUROPEO**	-0.00521	0.06403	0.02544	0.01356	0.02326	0.03195	-0.01167	-0.01140	-0.00188	0.00000			
**AFRICANO**	-0.00787	0.05473	0.01896	0.00838	0.01688	0.02446	-0.01137	-0.01090	-0.00360	-0.00542	0.00000		
**MEXICOAMERICANOS**	0.10238	0.24034	0.17453	0.14905	0.17083	0.18988	0.06116	0.05895	0.09538	0.06407	0.07433	0.00000	
**ASIATICOS**	0.23430	0.38133	0.31320	0.28494	0.31113	0.33621	0.17993	0.17647	0.21402	0.17496	0.18886	0.02571	0.00000

Table S18. rs7975232 (VDR) Fst p values

	MAZATECAS	PUREPECHAS	TARAHUMARAS	NAHUAS	MAYAS	TZELTALES	LACANDONES	HUICHOLES	MESTIZOS_JALISCO	EUROPEO	AFRICANO	MEXICOAMERICANOS	ASIATICOS
MAZATECAS	*												
PUREPECHAS	0.11296	*											
TARAHUMARAS	0.40323	0.42827	*										
NAHUAS	0.58489	0.22740	0.75389	*									
MAYAS	0.43154	0.45342	0.99990	0.70072	*								
TZELTALES	0.40996	0.83873	0.84190	0.66330	0.81655	*							
LACANDONES	0.69300	0.02633	0.15464	0.17810	0.14078	0.18563	*						
HUICHOLES	0.52421	0.01000	0.09425	0.13900	0.07395	0.07989	0.99990	*					
MESTIZOS_JALISCO	0.87962	0.01455	0.12969	0.29710	0.19731	0.21018	0.63776	0.62687	*				
EUROPEO	0.56747	0.00713	0.06465	0.11702	0.09148	0.13365	0.99990	0.99990	0.48411	*			
AFRICANO	0.65033	0.00792	0.10167	0.14781	0.10692	0.16820	0.99990	0.87447	0.60044	0.83408	*		
MEXICOAMERICANOS	0.00168	0.00000	0.00000	0.00000	0.00000	0.00020	0.01040	0.00792	0.00000	0.00149	0.00050	*	
ASIATICOS	0.00000	0.00000	0.00000	0.00000	0.00000	0.00000	0.00000	0.00000	0.00000	0.00000	0.00000	0.03366	*

Table S19. rs1800247 (BGLAP) pairwise Fst

	MAZATECAS	PUREPECHAS	TARAHUMARAS	NAHUAS	MAYAS	TZELTALES	LACANDONES	HUICHOLES	MESTIZOS_JALISCO	EUROPEO	AFRICANO	MEXICOAMERICANOS	ASIATICOS
MAZATECAS	0.00000												
PUREPECHAS	0.17118	0.00000											
TARAHUMARAS	-0.00891	0.22934	0.00000										
NAHUAS	-0.01679	0.15814	-0.00475	0.00000									
MAYAS	0.05307	0.01840	0.10750	0.05693	0.00000								

**TZELTALES**	0.26485	0.01956	0.30361	0.23213	0.08882	0.00000							
**LACANDONES**	0.20011	-0.01090	0.25150	0.18083	0.03765	0.00018	0.00000						
**HUICHOLES**	0.02448	0.03904	0.07220	0.03026	-0.01285	0.12314	0.06327	0.00000					
**MESTIZOS_JALISCO**	0.13429	-0.00259	0.20410	0.13188	0.00134	0.04102	0.00672	0.01416	0.00000				
**EUROPEO**	0.07028	0.01511	0.13145	0.07383	-0.00941	0.07190	0.03064	-0.00723	0.00186	0.00000			
**AFRICANO**	0.02435	0.04539	0.07330	0.03065	-0.00396	0.10871	0.06446	-0.01142	0.02485	0.00219	0.00000		
**MEXICOAMERICANOS**	0.04026	0.03138	0.09374	0.04559	-0.00916	0.09564	0.05006	-0.01259	0.01252	-0.00419	-0.00515	0.00000	
**ASIATICOS**	-0.01038	0.17106	-0.00819	-0.00580	0.07955	0.23534	0.19109	0.05205	0.15147	0.09707	0.05241	0.06821	0.00000

Table S20. rs1800247 (BGLAP) Fst p values

	**MAZATECAS**	**PUREPECHAS**	**TARAHUMARAS**	**NAHUAS**	**MAYAS**	**TZELTALES**	**LACANDONES**	**HUICHOLES**	**MESTIZOS_JALISCO**	**EUROPEO**	**AFRICANO**	**MEXICOAMERICANOS**	**ASIATICOS**
**MAZATECAS**	*												
**PUREPECHAS**	0.00139	*											
**TARAHUMARAS**	0.54163	0.00000	*										
**NAHUAS**	0.99990	0.00000	0.48767	*									
**MAYAS**	0.05782	0.20087	0.00436	0.02435	*								
**TZELTALES**	0.00040	0.31343	0.00000	0.00010	0.02505	*							
**LACANDONES**	0.00099	0.79269	0.00000	0.00020	0.11266	0.50856	*						
**HUICHOLES**	0.13721	0.07871	0.01218	0.06623	0.82427	0.00485	0.05257	*					
**MESTIZOS_JALISCO**	0.00059	0.42233	0.00000	0.00000	0.33561	0.04336	0.24512	0.11454	*				
**EUROPEO**	0.01000	0.12504	0.00010	0.00059	0.99990	0.01723	0.07385	0.51084	0.22889	*			
**AFRICANO**	0.11484	0.01970	0.00406	0.03307	0.51025	0.00178	0.01228	0.85229	0.01653	0.26730	*		
**MEXICOAMERICANOS**	0.05079	0.05069	0.00149	0.01188	0.85487	0.00287	0.02693	0.99990	0.07088	0.52262	0.66132	*	
**ASIATICOS**	0.71448	0.00000	0.75379	0.55499	0.00099	0.00000	0.00000	0.01139	0.00000	0.00000	0.00050	0.00000	*

Table S21. rs1544410 (VDR) Pairwise Fst

	MAZATECAS	PUREPECHAS	TARAHUMARAS	NAHUAS	MAYAS	TZELTALES	LACANDONES	HUICHOLES	MESTIZOS_JALISCO	EUROPEO	AFRICANO	MEXICOAMERICANOS	ASIATICOS
MAZATECAS	0.00000												
PUREPECHAS	0.03910	0.00000											
TARAHUMARAS	-0.10489	-0.14253	0.00000										
NAHUAS	-0.02048	0.03490	-0.09932	0.00000									
MAYAS	0.00500	-0.00743	-0.13252	0.00794	0.00000								
TZELTALES	-0.02661	0.05142	-0.10000	-0.02153	0.01205	0.00000							
LACANDONES	0.06671	-0.00487	0.00000	0.05342	0.00914	0.08085	0.00000						
HUICHOLES	-0.02019	0.00878	-0.12343	-0.01501	-0.01337	-0.01786	0.03732	0.00000					
MESTIZOS_JALISCO	0.05513	0.17096	0.01631	0.06489	0.13495	0.04460	0.17275	0.08355	0.00000				
EUROPEO	0.29133	0.40418	0.28057	0.30982	0.37501	0.27834	0.39624	0.32039	0.14494	0.00000			
AFRICANO	0.51472	0.61437	0.51877	0.53261	0.58932	0.50299	0.60513	0.54007	0.36618	0.07408	0.00000		
MEXICOAMERICANOS	0.66443	0.75636	0.67145	0.67898	0.73283	0.65396	0.74811	0.68772	0.51143	0.17709	0.02307	0.00000	
ASIATICOS	0.88725	0.92580	0.91116	0.88261	0.91409	0.88375	0.92868	0.90020	0.75341	0.41344	0.19807	0.09786	0.00000

Table S22. rs1544410 (VDR) Fst p values

	MAZATECAS	PUREPECHAS	TARAHUMARAS	NAHUAS	MAYAS	TZELTALES	LACANDONES	HUICHOLES	MESTIZOS_JALISCO	EUROPEO	AFRICANO	MEXICOAMERICANOS	ASIATICOS
MAZATECAS	*												
PUREPECHAS	0.19463	*											
TARAHUMARAS	0.99990	0.99990	*										
NAHUAS	0.99990	0.15177	0.99990	*									
MAYAS	0.44689	0.60935	0.99990	0.33442	*								
TZELTALES	0.99990	0.08465	0.99990	0.99990	0.32462	*							

**LACANDONES**	0.17632	0.99990	0.99990	0.11801	0.50332	0.06356	*						
**HUICHOLES**	0.99990	0.54529	0.99990	0.74913	0.61489	0.65835	0.17632	*					
**MESTIZOS_JALISCO**	0.15385	0.00218	0.90724	0.06772	0.00812	0.19018	0.00545	0.08613	*				
**EUROPEO**	0.00000	0.00000	0.15612	0.00000	0.00000	0.00000	0.00000	0.00000	0.00020	*			
**AFRICANO**	0.00000	0.00000	0.00792	0.00000	0.00000	0.00000	0.00000	0.00000	0.00000	0.00030	*		
**MEXICOAMERICANOS**	0.00000	0.00000	0.00099	0.00000	0.00000	0.00000	0.00000	0.00000	0.00000	0.00000	0.04148	*	
**ASIATICOS**	0.00000	0.00000	0.00000	0.00000	0.00000	0.00000	0.00000	0.00000	0.00000	0.00000	0.00000	0.00000	*

Table S23. rs1801197 (CALCR) pairwise Fst

	**MAZATECAS**	**PUREPECHAS**	**TARAHUMARAS**	**NAHUAS**	**MAYAS**	**TZELTALES**	**LACANDONES**	**HUICHOLES**	**MESTIZOS_JALISCO**	**EUROPEO**	**AFRICANO**	**MEXICOAMERICANOS**	**ASIATICOS**
**MAZATECAS**	0.00000												
**PUREPECHAS**	-0.01310	0.00000											
**TARAHUMARAS**	0.00103	-0.00017	0.00000										
**NAHUAS**	-0.00657	-0.00658	-0.00880	0.00000									
**MAYAS**	-0.01425	-0.01227	-0.00030	-0.00707	0.00000								
**TZELTALES**	-0.01963	-0.01647	0.00394	-0.00611	-0.01788	0.00000							
**LACANDONES**	-0.01197	-0.00781	0.01946	0.00688	-0.00951	-0.02000	0.00000						
**HUICHOLES**	-0.01321	-0.01249	-0.00966	-0.01293	-0.01319	-0.01424	-0.00282	0.00000					
**MESTIZOS_JALISCO**	-0.00356	-0.00359	-0.00584	-0.00713	-0.00407	-0.00302	0.01001	-0.00994	0.00000				
**EUROPEO**	0.11704	0.12649	0.19759	0.16909	0.12375	0.09668	0.07817	0.15248	0.16698	0.00000			
**AFRICANO**	-0.00265	0.00211	0.03326	0.01940	0.00025	-0.01197	-0.01071	0.00889	0.02248	0.06683	0.00000		
**MEXICOAMERICANOS**	-0.00963	-0.00613	0.01642	0.00571	-0.00763	-0.01636	-0.01180	-0.00284	0.00876	0.09143	-0.00459	0.00000	
**ASIATICOS**	0.25848	0.23723	0.15674	0.18492	0.24581	0.29706	0.31553	0.21637	0.16345	0.51229	0.29399	0.26933	0.00000

Table S24. rs1801197 (CALCR) Fst p values

	MAZATECAS	PUREPECHAS	TARAHUMARAS	NAHUAS	MAYAS	TZELTALES	LACANDONES	HUICHOLES	MESTIZOS_JALISCO	EUROPEO	AFRICANO	MEXICOAMERICANOS	ASIATICOS
**MAZATECAS**	*												
**PUREPECHAS**	0.99990+	*											
**TARAHUMARAS**	0.08306	0.10692	*										
**NAHUAS**	0.22810	0.27611	0.63390	*									
**MAYAS**	0.99990	0.99990	0.09643	0.26324	*								
**TZELTALES**	0.65588	0.65766	0.09217	0.16236	0.65558	*							
**LACANDONES**	0.24453	0.15206	0.00228	0.00980	0.12959	0.39798	*						
**HUICHOLES**	0.46847	0.74339	0.41045	0.77576	0.48738	0.37165	0.05277	*					
**MESTIZOS_JALISCO**	0.20404	0.25225	0.53658	0.99990	0.22265	0.12524	0.00881	0.79893	*				
**EUROPEO**	0.00000	0.00000	0.00000	0.00000	0.00000	0.00347	0.00050	0.00000	0.00000	*			
**AFRICANO**	0.41521	0.23186	0.01703	0.04861	0.27928	0.70340	0.86506	0.19077	0.00366	0.00040	*		
**MEXICOAMERICANOS**	0.72171	0.50520	0.06435	0.15929	0.60400	0.99990	0.85675	0.37511	0.04485	0.00020	0.65132	*	
**ASIATICOS**	0.00000	0.00000	0.00000	0.00000	0.00000	0.00000	0.00000	0.00000	0.00000	0.00000	0.00000	0.00000	*

Table S24. rs1800012 (COL1A1) pairwise Fst

	MAZATECAS	PUREPECHAS	TARAHUMARAS	NAHUAS	MAYAS	TZELTALES	LACANDONES	HUICHOLES	MESTIZOS_JALISCO	EUROPEO	AFRICANO	MEXICOAMERICANOS	ASIATICOS
**MAZATECAS**	0.00000												
**PUREPECHAS**	0.00000	0.00000											
**TARAHUMARAS**	0.00447	0.01085	0.00000										
**NAHUAS**	-0.00452	0.00000	-0.00668	0.00000									
**MAYAS**	0.01169	0.02084	-0.01125	-0.00322	0.00000								
**TZELTALES**	0.00000	0.00000	-0.00287	-0.01056	0.00238	0.00000							

**LACANDONES**	0.00000	0.00000	0.00351	-0.00526	0.01041	0.00000	0.00000						
**HUICHOLES**	0.00000	0.00000	0.00351	-0.00526	0.01041	0.00000	0.00000	0.00000					
**MESTIZOS_JALISCO**	0.04957	0.05796	0.02893	0.04260	0.01866	0.04101	0.04839	0.04839	0.00000				
**EUROPEO**	0.11930	0.13764	0.10053	0.11898	0.08229	0.10366	0.11694	0.11694	0.02840	0.00000			
**AFRICANO**	0.01706	0.02350	-0.00248	0.00742	-0.00780	0.00974	0.01610	0.01610	0.01493	0.08800	0.00000		
**MEXICOAMERICANOS**	0.11923	0.14077	0.09864	0.11936	0.07864	0.10112	0.11647	0.11647	0.02208	-0.00644	0.08400	0.00000	
**ASIATICOS**	-0.00702	-0.00382	0.00418	-0.00585	0.01328	-0.01212	-0.00760	-0.00760	0.06513	0.16748	0.02263	0.17511	0.00000

Table S25. rs1800012 (COL1A1) Fst p values

	**MAZATECAS**	**PUREPECHAS**	**TARAHUMARAS**	**NAHUAS**	**MAYAS**	**TZELTALES**	**LACANDONES**	**HUICHOLES**	**MESTIZOS_JALISCO**	**EUROPEO**	**AFRICANO**	**MEXICOAMERICANOS**	**ASIATICOS**
**MAZATECAS**	*												
**PUREPECHAS**	0.99990	*											
**TARAHUMARAS**	0.51124	0.24196	*										
**NAHUAS**	0.99990	0.99990	0.61687	*									
**MAYAS**	0.49589	0.17325	0.99990	0.57212	*								
**TZELTALES**	0.99990	0.99990	0.58133	0.99990	0.53559	*							
**LACANDONES**	0.99990	0.99990	0.52391	0.99990	0.49312	0.99990	*						
**HUICHOLES**	0.99990	0.99990	0.52737	0.99990	0.49530	0.99990	0.99990	*					
**MESTIZOS_JALISCO**	0.01663	0.00693	0.04307	0.01000	0.09623	0.06900	0.03148	0.02782	*				
**EUROPEO**	0.00218	0.00010	0.00119	0.00030	0.00535	0.01426	0.00327	0.00356	0.02435	*			
**AFRICANO**	0.18236	0.05108	0.49520	0.25908	0.72171	0.34690	0.18978	0.18830	0.06277	0.00010	*		
**MEXICOAMERICANOS**	0.00119	0.00000	0.00030	0.00010	0.00822	0.00822	0.00168	0.00277	0.04198	0.89100	0.00020	*	
**ASIATICOS**	0.99990	0.99990	0.26126	0.99990	0.17464	0.99990	0.99990	0.99990	0.00000	0.00000	0.02742	0.00000	*

Table S26. rs2073617 (TNFRS11B) pairwise Fst

	MAZATECAS	PUREPECHAS	TARAHUMARAS		MAYAS	TZELTALES	LACANDONES	HUICHOLES	MESTIZOS_JALISCO	EUROPEO	AFRICANO	MEXICOAMERICANOS	ASIATICOS
**MAZATECAS**	0.00000												
**PUREPECHAS**	-0.01000	0.00000											
**TARAHUMARAS**	0.00887	-0.00311	0.00000										
**NAHUAS**	-0.00502	0.01108	0.04315	0.00000									
**MAYAS**	0.00931	-0.00334	-0.01192	0.04362	0.00000								
**TZELTALES**	-0.01723	-0.01839	-0.00986	0.00320	-0.00999	0.00000							
**LACANDONES**	0.00327	-0.00757	-0.01390	0.03537	-0.01545	-0.01444	0.00000						
**HUICHOLES**	0.03529	0.06814	0.12311	0.00642	0.12225	0.05587	0.10844	0.00000					
**MESTIZOS_JALISCO**	0.01339	0.03491	0.07084	-0.00243	0.07134	0.02684	0.06299	-0.00510	0.00000				
**EUROPEO**	0.12866	0.16919	0.22594	0.08346	0.22364	0.15510	0.21003	0.01858	0.05442	0.00000			
**AFRICANO**	0.53114	0.56651	0.61957	0.46165	0.62244	0.56926	0.61417	0.36534	0.38693	0.20449	0.00000		
**MEXICOAMERICANOS**	0.02229	0.04820	0.09127	0.00108	0.09106	0.03862	0.08068	-0.01064	-0.00476	0.03804	0.37763	0.00000	
**ASIATICOS**	0.17603	0.21871	0.27656	0.12595	0.27407	0.20436	0.26038	0.04772	0.09153	-0.00035	0.15200	0.07158	0.00000

Table S27. rs2073617 (TNFRS11B) Fst p values

	MAZATECAS	PUREPECHAS	TARAHUMARAS	NAHUAS	MAYAS	TZELTALES	LACANDONES	HUICHOLES	MESTIZOS_JALISCO	EUROPEO	AFRICANO	MEXICOAMERICANOS	ASIATICOS
**MAZATECAS**	*												
**PUREPECHAS**	0.65845	*											
**TARAHUMARAS**	0.18147	0.39600	*										
**NAHUAS**	0.45302	0.14781	0.02039	*									
**MAYAS**	0.29868	0.40451	0.99990	0.03257	*								
**TZELTALES**	0.81645	0.99990	0.61885	0.30601	0.65102	*							

**LACANDONES**	0.32036	0.47391	0.99990	0.06643	0.99990	0.60964	*						
**HUICHOLES**	0.07207	0.01069	0.00139	0.28225	0.00386	0.09851	0.00564	*					
**MESTIZOS_JALISCO**	0.13771	0.02277	0.00079	0.45659	0.00307	0.11722	0.01178	0.54787	*				
**EUROPEO**	0.00030	0.00000	0.00000	0.00069	0.00000	0.00119	0.00000	0.13543	0.00069	*			
**AFRICANO**	0.00000	0.00000	0.00000	0.00000	0.00000	0.00000	0.00000	0.00000	0.00000	0.00000	*		
**MEXICOAMERICANOS**	0.11029	0.01663	0.00119	0.32294	0.00297	0.10217	0.00535	0.75686	0.73854	0.01525	0.00000	*	
**ASIATICOS**	0.00000	0.00000	0.00000	0.00000	0.00000	0.00020	0.00000	0.02940	0.00000	0.34848	0.00000	0.00059	*

Table S28. rs700518 (CYP19A1) pairwise Fst

	**MAZATECAS**	**PUREPECHAS**	**TARAHUMARAS**	**NAHUAS**	**MAYAS**	**TZELTALES**	**LACANDONES**	**HUICHOLES**	**MESTIZOS_JALISCO**	**EUROPEO**	**AFRICANO**	**MEXICOAMERICANOS**	**ASIATICOS**
**MAZATECAS**	0.00000												
**PUREPECHAS**	0.00000	0.00000											
**TARAHUMARAS**	0.01626	0.02888	0.00000										
**NAHUAS**	0.00000	0.00000	0.03052	0.00000									
**MAYAS**	0.00000	0.00000	0.02419	0.00000	0.00000								
**TZELTALES**	0.14295	0.20332	0.06569	0.21187	0.17938	0.00000							
**LACANDONES**	-0.00156	0.01011	-0.00724	0.01159	0.00583	0.09456	0.00000						
**HUICHOLES**	0.00000	0.00000	0.02089	0.00000	0.00000	0.16342	0.00278	0.00000					
**MESTIZOS_JALISCO**	0.00000	0.00000	0.05645	0.00000	0.00000	0.34162	0.03458	0.00000	0.00000				
**EUROPEO**	0.34987	0.39017	0.33512	0.39595	0.37406	0.16799	0.32973	0.36342	0.48571	0.00000			
**AFRICANO**	0.83068	0.85191	0.81683	0.85478	0.84367	0.71642	0.81914	0.83803	0.89329	0.33500	0.00000		
**MEXICOAMERICANOS**	0.57160	0.61930	0.56931	0.62583	0.60067	0.39124	0.55455	0.58801	0.71624	0.07295	0.13066	0.00000	
**ASIATICOS**	0.37717	0.41456	0.36315	0.41994	0.39959	0.20002	0.35803	0.38971	0.50387	-0.00304	0.29044	0.04893	0.00000

Table S29. rs700518 (CYP19A1) Fst p values

	MAZATECAS	PUREPECHAS	TARAHUMARAS	NAHUAS	MAYAS	TZELTALES	LACANDONES	HUICHOLES	MESTIZOS_JALISCO	EUROPEO	AFRICANO	MEXICOAMERICANOS	ASIATICOS
MAZATECAS	*												
PUREPECHAS	0.99990	*											
TARAHUMARAS	0.29413	0.11989	*										
NAHUAS	0.99990	0.99990	0.12029	*									
MAYAS	0.99990	0.99990	0.13355	0.99990	*								
TZELTALES	0.08554	0.02723	0.08029	0.02247	0.04653	*							
LACANDONES	0.99990	0.38085	0.64419	0.35551	0.42293	0.16375	*						
HUICHOLES	0.99990	0.99990	0.16058	0.99990	0.99990	0.06039	0.46283	*					
MESTIZOS_JALISCO	0.99990	0.99990	0.00931	0.99990	0.99990	0.00416	0.20147	0.99990	*				
EUROPEO	0.00000	0.00000	0.00000	0.00000	0.00000	0.00040	0.00000	0.00000	0.00000	*			
AFRICANO	0.00000	0.00000	0.00000	0.00000	0.00000	0.00000	0.00000	0.00000	0.00000	0.00000	*		
MEXICOAMERICANOS	0.00000	0.00000	0.00000	0.00000	0.00000	0.00000	0.00000	0.00000	0.00000	0.00020	0.00000	*	
ASIATICOS	0.00000	0.00000	0.00000	0.00000	0.00000	0.00010	0.00000	0.00000	0.00000	0.53628	0.00000	0.00198	*

Table S30. Rs724449 (PTHR1) pairwise Fst

	MAZATECAS	PUREPECHAS	TARAHUMARAS	NAHUAS	MAYAS	TZELTALES	LACANDONES	HUICHOLES	MESTIZOS_JALISCO	EUROPEO	AFRICANO	MEXICOAMERICANOS	ASIATICOS
MAZATECAS	0.00000												
PUREPECHAS	-0.01053	0.00000											
TARAHUMARAS	-0.00893	-0.01400	0.00000										
NAHUAS	-0.00569	0.01248	0.02026	0.00000									
MAYAS	-0.01450	-0.00506	-0.00183	-0.00823	0.00000								
TZELTALES	0.04514	0.08032	0.09706	0.00638	0.03506	0.00000							

**LACANDONES**	0.08851	0.12820	0.14830	0.03838	0.07546	-0.01581	0.00000						
**HUICHOLES**	-0.01460	-0.01406	-0.01574	0.00603	-0.00973	0.07053	0.11822	0.00000					
**MESTIZOS_JALISCO**	-0.00995	-0.00102	0.00191	-0.00305	-0.00864	0.04103	0.08025	-0.00553	0.00000				
**EUROPEO**	-0.01033	-0.00792	-0.00852	0.00740	-0.00625	0.06750	0.11216	-0.01116	-0.00200	0.00000			
**AFRICANO**	0.29214	0.33323	0.36773	0.21183	0.26848	0.09768	0.05559	0.33022	0.24849	0.29818	0.00000		
**MEXICOAMERICANOS**	-0.01038	0.00113	0.00544	-0.00773	-0.01014	0.02993	0.06760	-0.00392	-0.00536	-0.00101	0.24428	0.00000	
**ASIATICOS**	0.04322	0.07460	0.08935	0.00920	0.03435	-0.01466	-0.00147	0.06587	0.03988	0.06336	0.10611	0.02998	0.00000

Table S31. rs724449 (PTHR1) Fst p values

	**MAZATECAS**	**PUREPECHAS**	**TARAHUMARAS**	**NAHUAS**	**MAYAS**	**TZELTALES**	**LACANDONES**	**HUICHOLES**	**MESTIZOS_JALISCO**	**EUROPEO**	**AFRICANO**	**MEXICOAMERICANOS**	**ASIATICOS**
**MAZATECAS**	*												
**PUREPECHAS**	0.74131	*											
**TARAHUMARAS**	0.57272	0.85516	*										
**NAHUAS**	0.49342	0.19770	0.14949	*									
**MAYAS**	0.87229	0.50985	0.35274	0.60420	*								
**TZELTALES**	0.09247	0.01396	0.01178	0.27680	0.07455	*							
**LACANDONES**	0.02841	0.00287	0.00495	0.08960	0.01673	0.66419	*						
**HUICHOLES**	0.85853	0.99990	0.85605	0.29769	0.59677	0.03069	0.01129	*					
**MESTIZOS_JALISCO**	0.88912	0.37947	0.29957	0.43124	0.99990	0.05504	0.00376	0.56846	*				
**EUROPEO**	0.88348	0.80546	0.65261	0.23364	0.59232	0.02683	0.00218	0.99990	0.49906	*			
**AFRICANO**	0.00000	0.00000	0.00000	0.00000	0.00000	0.00525	0.01851	0.00000	0.00000	0.00000	*		
**MEXICOAMERICANOS**	0.73300	0.28601	0.21691	0.63122	0.87041	0.05762	0.00772	0.40065	0.82130	0.41135	0.00000	*	
**ASIATICOS**	0.03505	0.00158	0.00396	0.17315	0.03000	0.86160	0.38858	0.00990	0.00228	0.00079	0.00000	0.01307	*

Table S32. rs1800795 (IL6) pairwise Fst

	MAZATECAS	PUREPECHAS	TARAHUMARAS	NAHUAS	MAYAS	TZELTALES	LACANDONES	HUICHOLES	MESTIZOS_JALISCO	EUROPEO	MEXICOAMERICANOS
MAZATECAS	0.00000										
PUREPECHAS	0.00000	0.00000									
TARAHUMARAS	0.00000	0.00000	0.00000								
NAHUAS	0.00000	0.00000	0.00000	0.00000							
MAYAS	0.00000	0.00000	0.00000	0.00000	0.00000						
TZELTALES	0.00000	0.00000	0.00000	0.00000	0.00000	0.00000					
LACANDONES	0.00000	0.00000	0.00000	0.00000	0.00000	0.00000	0.00000				
HUICHOLES	0.00000	0.00000	0.00000	0.00000	0.00000	0.00000	0.00000	0.00000			
MESTIZOS_JALISCO	0.00000	0.00000	0.00000	0.00000	0.00000	0.00000	0.00000	0.00000	0.00000		
EUROPEO	0.39509	0.43063	0.43640	0.43257	0.41456	0.37356	0.39282	0.39734	0.54685	0.00000	
MEXICOAMERICANOS	0.81811	0.84169	0.84522	0.84288	0.83141	0.80245	0.81649	0.81969	0.89966	0.25093	0.00000

Table S33. rs1800795 (IL6) Fst p values

	MAZATECAS	PUREPECHAS	TARAHUMARAS	NAHUAS	MAYAS	TZELTALES	LACANDONES	HUICHOLES	MESTIZOS_JALISCO	EUROPEO	MEXICOAMERICANOS
MAZATECAS	*										
PUREPECHAS	0.99990	*									
TARAHUMARAS	0.99990	0.99990	*								
NAHUAS	0.99990	0.99990	0.99990	*							
MAYAS	0.99990	0.99990	0.99990	0.99990	*						
TZELTALES	0.99990	0.99990	0.99990	0.99990	0.99990	*					
LACANDONES	0.99990	0.99990	0.99990	0.99990	0.99990	0.99990	*				
HUICHOLES	0.99990	0.99990	0.99990	0.99990	0.99990	0.99990	0.99990	*			
MESTIZOS_JALISCO	0.99990	0.99990	0.99990	0.99990	0.99990	0.99990	0.99990	0.99990	*		

**EUROPEO**	0.00000	0.00000	0.00000	0.00000	0.00000	0.00000	0.00000	0.00000	0.00000	*	
**MEXICOAMERICANOS**	0.00000	0.00000	0.00000	0.00000	0.00000	0.00000	0.00000	0.00000	0.00000	0.00000	*

Table S34. rs731236 (VDR) pairwise Fst

	**MAZATECAS**	**PUREPECHAS**	**TARAHUMARAS**	**NAHUAS**	**MAYAS**	**TZELTALES**	**LACANDONES**	**HUICHOLES**	**MESTIZOS_JALISCO**	**EUROPEO**	**AFRICANO**	**MEXICOAMERICANOS**	**ASIATICOS**
**MAZATECAS**	0.00000												
**PUREPECHAS**	-0.01395	0.00000											
**TARAHUMARAS**	-0.01460	-0.01147	0.00000										
**NAHUAS**	0.04005	0.05007	0.03639	0.00000									
**MAYAS**	-0.00018	0.00475	-0.00298	0.00262	0.00000								
**TZELTALES**	0.01819	0.02673	0.01297	-0.01487	-0.01558	0.00000							
**LACANDONES**	-0.01274	-0.01038	-0.00771	0.06141	0.01710	0.04560	0.00000						
**HUICHOLES**	-0.01748	-0.01442	-0.01555	0.03558	-0.00300	0.01352	-0.01197	0.00000					
**MESTIZOS_JALISCO**	0.08949	0.10073	0.08732	0.01150	0.04896	0.02069	0.10696	0.08518	0.00000				
**EUROPEO**	0.35968	0.38765	0.36523	0.25350	0.31401	0.26268	0.37566	0.35295	0.17908	0.00000			
**AFRICANO**	0.58909	0.61485	0.59504	0.49536	0.55054	0.50372	0.60189	0.58310	0.41572	0.08352	0.00000		
**MEXICOAMERICANOS**	0.71057	0.73413	0.71567	0.61625	0.67256	0.62962	0.72299	0.70485	0.52351	0.16824	0.01367	0.00000	
**ASIATICOS**	0.91820	0.92008	0.91470	0.84733	0.89213	0.88339	0.92688	0.91663	0.73151	0.42347	0.19630	0.11603	0.00000

Table S35. rs731236 (VDR) Fst p values

	MAZATECAS	PUREPECHAS	TARAHUMARAS	NAHUAS	MAYAS	TZELTALES	LACANDONES	HUICHOLES	MESTIZOS_JALISCO	EUROPEO	AFRICANO	MEXICOAMERICANOS	ASIATICOS
**MAZATECAS**	*												
**PUREPECHAS**	0.99990	*											
**TARAHUMARAS**	0.99990	0.99990	*										
**NAHUAS**	0.14009	0.05415	0.10375	*									
**MAYAS**	0.51104	0.33155	0.53955	0.39422	*								
**TZELTALES**	0.19463	0.18721	0.22097	0.80230	0.75418	*							
**LACANDONES**	0.99990	0.65845	0.63756	0.07098	0.30225	0.14375	*						
**HUICHOLES**	0.99990	0.99990	0.99990	0.14286	0.52836	0.38165	0.99990	*					
**MESTIZOS_JALISCO**	0.00327	0.00010	0.00089	0.16404	0.01960	0.13543	0.00119	0.00426	*				
**EUROPEO**	0.00000	0.00000	0.00000	0.00000	0.00000	0.00000	0.00000	0.00000	0.00000	*			
**AFRICANO**	0.00000	0.00000	0.00000	0.00000	0.00000	0.00000	0.00000	0.00000	0.00000	0.00000	*		
**MEXICOAMERICANOS**	0.00000	0.00000	0.00000	0.00000	0.00000	0.00000	0.00000	0.00000	0.00000	0.00000	0.07940	*	
**ASIATICOS**	0.00000	0.00000	0.00000	0.00000	0.00000	0.00000	0.00000	0.00000	0.00000	0.00000	0.00000	0.00000	*

Table S36. rs180469 (TGFB1) pairwise Fst

	MAZATECAS	PUREPECHAS	TARAHUMARAS	NAHUAS	MAYAS	TZELTALES	LACANDONES	HUICHOLES	MESTIZOS_JALISCO	EUROPEO	AFRICANO	MEXICOAMERICANOS	ASIATICOS
MAZATECAS	0.00000												
PUREPECHAS	0.32158	0.00000											
TARAHUMARAS	0.23423	0.00182	0.00000										
NAHUAS	0.27839	-0.00714	-0.00737	0.00000									
MAYAS	0.07033	0.09780	0.04021	0.06703	0.00000								
TZELTALES	0.08155	0.08361	0.02187	0.04916	-0.01833	0.00000							
LACANDONES	-0.01460	0.28314	0.19728	0.24031	0.04588	0.05587	0.00000						
HUICHOLES	0.07289	0.09562	0.03602	0.06340	-0.01542	-0.02042	0.04816	0.00000					
MESTIZOS_JALISCO	0.03319	0.12265	0.07565	0.09916	-0.00055	0.00157	0.01519	-0.00025	0.00000				
EUROPEO	0.01921	0.14924	0.09519	0.12226	0.00517	0.00918	0.00457	0.00594	-0.00391	0.00000			
AFRICANO	0.08080	0.08193	0.03781	0.05901	-0.00988	-0.01418	0.05441	-0.01123	0.00399	0.01012	0.00000		
MEXICOAMERICANOS	-0.01227	0.27166	0.20627	0.23991	0.06979	0.08038	-0.00994	0.07217	0.03626	0.02286	0.07971	0.00000	
ASIATICOS	-0.00305	0.31573	0.25681	0.28759	0.11809	0.13051	0.00760	0.12087	0.07826	0.05990	0.13029	0.00155	0.00000

Table S37. rs180469 (TGFB1) Fst p values

	MAZATECAS	PUREPECHAS	TARAHUMARAS	NAHUAS	MAYAS	TZELTALES	LACANDONES	HUICHOLES	MESTIZOS_JALISCO	EUROPEO	AFRICANO	MEXICOAMERICANOS	ASIATICOS
MAZATECAS	*												
PUREPECHAS	0.00000	*											
TARAHUMARAS	0.00000	0.32314	*										
NAHUAS	0.00000	0.78626	0.61350										
MAYAS	0.01089	0.00248	0.03109	0.00663	*								

**TZELTALES**	0.01841	0.02901	0.12791	0.05683	0.78893	*							
**LACANDONES**	0.81338	0.00000	0.00000	0.00000	0.01277	0.01436	*						
**HUICHOLES**	0.01673	0.00337	0.04148	0.00931	0.99990	0.99990	0.01673	*					
**MESTIZOS_JALISCO**	0.01742	0.00000	0.00010	0.00010	0.28730	0.25364	0.04871	0.24562+	*				
**EUROPEO**	0.11484	0.00000	0.00020	0.00000	0.25819	0.23592	0.22255	0.23592	0.63281	*			
**AFRICANO**	0.00574	0.00119	0.02525	0.00653	0.99990	0.83942	0.01238	0.99990	0.15008	0.12405	*		
**MEXICOAMERICANOS**	0.99990	0.00000	0.00000	0.00000	0.00495	0.01327	0.72250	0.00733	0.00277	0.03930	0.00059	*	
**ASIATICOS**	0.45293	0.00000	0.00000	0.00000	0.00050	0.00228	0.21285	0.00059	0.00000	0.00040	0.00000	0.31403	*

Table S38. rs1061624 (TNFRSF1B) pairwise Fst

	**MAZATECAS**	**PUREPECHAS**	**TARAHUMARAS**	**NAHUAS**	**MAYAS**	**TZELTALES**	**LACANDONES**	**HUICHOLES**	**MESTIZOS_JALISCO**	**EUROPEO**	**AFRICANO**	**MEXICOAMERICANOS**	**ASIATICOS**
**MAZATECAS**	0.00000												
**PUREPECHAS**	-0.00677	0.00000											
**TARAHUMARAS**	-0.01180	-0.00865	0.00000										
**NAHUAS**	-0.00677	-0.01031	-0.00865	0.00000									
**MAYAS**	-0.00641	-0.01202	-0.00926	-0.01202	0.00000								
**TZELTALES**	0.02816	0.00286	0.01596	0.00286	-0.00242	0.00000							
**LACANDONES**	0.02271	0.00070	0.01205	0.00070	-0.00403	-0.02061	0.00000						
**HUICHOLES**	-0.00084	-0.01153	-0.00607	-0.01153	-0.01445	-0.01121	-0.01131	0.00000					
**MESTIZOS_JALISCO**	-0.00125	-0.00696	-0.00418	-0.00696	-0.00901	0.00229	0.00078	-0.00937	0.00000				
**EUROPEO**	0.01154	0.04269	0.02618	0.04269	0.04655	0.10955	0.09951	0.06024	0.05181	0.00000			

**AFRICANO**	0.11711	0.16992	0.14149	0.16992	0.17914	0.27193	0.25572	0.20189	0.17699	0.03912	0.00000		
**MEXICOAMERICANOS**	-0.01157	-0.00055	-0.00658	-0.00055	0.00012	0.03654	0.03079	0.00636	0.00525	0.01190	0.10941	0.00000	
**ASIATICOS**	-0.00706	-0.00731	-0.00740	-0.00731	-0.00846	0.01123	0.00826	-0.00664	-0.00343	0.03674	0.15424	-0.00134	0.00000

Table S39. rs1061624 (TNFRSF1B) Fst p values

	**MAZATECAS**	**PUREPECHAS**	**TARAHUMARAS**	**NAHUAS**	**MAYAS**	**TZELTALES**	**LACANDONES**	**HUICHOLES**	**MESTIZOS_JALISCO**	**EUROPEO**	**AFRICANO**	**MEXICOAMERICANOS**	**ASIATICOS**
**MAZATECAS**	*												
**PUREPECHAS**	0.45698	*											
**TARAHUMARAS**	0.87298	0.74171	*										
**NAHUAS**	0.51332	0.99990	0.77913	*									
**MAYAS**	0.51539	0.99990	0.66290	0.99990	*								
**TZELTALES**	0.15513	0.25225	0.19285	0.34541	0.45520	*							
**LACANDONES**	0.15523	0.26661	0.20176	0.34096	0.51935	0.99990	*						
**HUICHOLES**	0.33125	0.68142	0.48391	0.71993	0.85863	0.50074	0.70072	*					
**MESTIZOS_JALISCO**	0.39471	0.89892	0.53787	0.89793	0.99990	0.29888	0.38323	0.76181	*				
**EUROPEO**	0.12296	0.00178	0.02396	0.00743	0.00871	0.00089	0.00050	0.00248	0.00059	*			
**AFRICANO**	0.00040	0.00000	0.00000	0.00000	0.00000	0.00000	0.00000	0.00000	0.00000	0.00218	*		
**MEXICOAMERICANOS**	0.99990	0.29849	0.67924	0.34115	0.38838	0.09672	0.08613	0.24800	0.18978	0.06504	0.00010	*	
**ASIATICOS**	0.65469	0.89169	0.90021	0.90050	0.78032	0.20137	0.22176	0.52737	0.62281	0.00158	0.00000	0.41352	*

Table S40. rs3397 (TNFRSF1B) pairwise Fst

	MAZATECAS	PUREPECHAS	TARAHUMARAS	NAHUAS	MAYAS	TZELTALES	LACANDONES	HUICHOLES	MESTIZOS_JALISCO	EUROPEO	AFRICANO	MEXICOAMERICANOS	ASIATICOS
**MAZATECAS**	0.00000												
**PUREPECHAS**	0.09202	0.00000											
**TARAHUMARAS**	0.15813	0.00235	0.00000										
**NAHUAS**	0.03930	0.00187	0.03989	0.00000									
**MAYAS**	0.15075	-0.00328	-0.01159	0.03072	0.00000								
**TZELTALES**	0.20945	0.00370	-0.01721	0.05180	-0.01674	0.00000							
**LACANDONES**	-0.01577	0.08023	0.14411	0.03070	0.13603	0.18951	0.00000						
**HUICHOLES**	-0.01243	0.06158	0.12254	0.01715	0.11352	0.15993	-0.01525	0.00000					
**MESTIZOS_JALISCO**	0.11511	-0.00241	-0.00548	0.02424	-0.00816	-0.00910	0.10533	0.08875	0.00000				
**EUROPEO**	0.22855	0.05086	0.01012	0.10754	0.01471	-0.00435	0.21568	0.19557	0.02732	0.00000			
**AFRICANO**	0.72101	0.53379	0.44814	0.60573	0.47012	0.44761	0.71204	0.69667	0.45305	0.32089	0.00000		
**MEXICOAMERICANOS**	0.12617	-0.00385	-0.00752	0.02455	-0.01009	-0.01134	0.11436	0.09547	-0.00580	0.02407	0.46931	0.00000	
**ASIATICOS**	0.47461	0.27095	0.18977	0.35030	0.20336	0.16415	0.46209	0.44314	0.22495	0.10175	0.08363	0.22016	0.00000

Table S41. rs3397 (TNFRSF1B) Fst p values

	MAZATECAS	PUREPECHAS	TARAHUMARAS	NAHUAS	MAYAS	TZELTALES	LACANDONES	HUICHOLES	MESTIZOS_JALISCO	EUROPEO	AFRICANO	MEXICOAMERICANOS	ASIATICOS
**MAZATECAS**	*												
**PUREPECHAS**	0.00267	*											
**TARAHUMARAS**	0.00010	0.27740	*										
**NAHUAS**	0.04079	0.25205	0.02505	*									

**MAYAS**	0.00069	0.46471	0.86031	0.07861	*								
**TZELTALES**	0.00059	0.25463	0.83091	0.04326	0.83813	*							
**LACANDONES**	0.99990	0.00554	0.00020	0.09692	0.00158	0.00218	*						
**HUICHOLES**	0.78854	0.02069	0.00139	0.19820	0.00891	0.01020	0.99990	*					
**MESTIZOS_JALISCO**	0.00059	0.41738	0.68112	0.04782	0.88694	0.58212	0.00040	0.00208	*				
**EUROPEO**	0.00000	0.00465	0.11712	0.00010	0.10613	0.45075	0.00000	0.00000	0.01148	*			
**AFRICANO**	0.00000	0.00000	0.00000	0.00000	0.00000	0.00000	0.00000	0.00000	0.00000	0.00000	*		
**MEXICOAMERICANOS**	0.00099	0.46134	0.77695	0.08237	0.88833	0.71528	0.00228	0.01049	0.99990	0.04099	0.00000	*	
**ASIATICOS**	0.00000	0.00000	0.00000	0.00000	0.00000	0.00040	0.00000	0.00000	0.00000	0.00000	0.00000	0.00000	*

Table S42. rs9340799 (ESR1) pairwise Fst

	**MAZATECAS**	**PUREPECHAS**	**TARAHUMARAS**	**NAHUAS**	**MAYAS**	**TZELTALES**	**LACANDONES**	**HUICHOLES**	**MESTIZOS_JALISCO**	**EUROPEO**	**AFRICANO**	**MEXICOAMERICANOS**	**ASIATICOS**
**MAZATECAS**	0.00000												
**PUREPECHAS**	0.00286	0.00000											
**TARAHUMARAS**	-0.00282	0.03839	0.00000										
**NAHUAS**	-0.00918	0.02348	-0.00895	0.00000									
**MAYAS**	-0.01379	0.01408	-0.00816	-0.01180	0.00000								
**TZELTALES**	0.01592	0.08874	-0.01048	-0.00158	0.00378	0.00000							
**LACANDONES**	0.11770	0.21875	0.06521	0.08787	0.09756	0.01603	0.00000						
**HUICHOLES**	-0.00933	0.03112	-0.01334	-0.01332	-0.01351	-0.01004	0.06850	0.00000					
**MESTIZOS_JALISCO**	0.07210	0.13288	0.03587	0.05085	0.05813	0.00228	-0.00654	0.03864	0.00000				
**EUROPEO**	0.07177	0.13799	0.03435	0.04986	0.05725	0.00036	-0.00686	0.03703	-0.00498	0.00000			
**AFRICANO**	0.03124	0.08566	0.00505	0.01492	0.02018	-0.01454	0.01686	0.00607	0.00541	0.00371	0.00000		
**MEXICOAMERICANOS**	0.00985	0.05750	-0.00664	-0.00128	0.00173	-0.01527	0.04315	-0.00761	0.02140	0.01963	-0.00215	0.00000	
**ASIATICOS**	0.01125	0.05579	-0.00515	0.00030	0.00330	-0.01422	0.04442	-0.00604	0.02232	0.02057	-0.00126	-0.00640	0.00000

Table S43. rs9340799 (ESR1) Fst p values

	MAZATECAS	PUREPECHAS	TARAHUMARAS	NAHUAS	MAYAS	TZELTALES	LACANDONES	HUICHOLES	MESTIZOS_JALISCO	EUROPEO	AFRICANO	MEXICOAMERICANOS	ASIATICOS
MAZATECAS	*												
PUREPECHAS	0.37323	*											
TARAHUMARAS	0.51638	0.05544	*										
NAHUAS	0.67667	0.14424	0.86605	*									
MAYAS	0.80368	0.20097	0.68558	0.84655	*								
TZELTALES	0.30066	0.02643	0.66013	0.52480	0.34294	*							
LACANDONES	0.00990	0.00010	0.03297	0.01703	0.01277	0.31829	*						
HUICHOLES	0.57727	0.09118	0.99990	0.99990	0.80081	0.60281	0.04455	*					
MESTIZOS_JALISCO	0.00059	0.00000	0.00792	0.00119	0.00188	0.28779	0.60133	0.01188	*				
EUROPEO	0.00842	0.00000	0.03604	0.01297	0.01436	0.37095	0.64697	0.04722	0.99990	*			
AFRICANO	0.06940	0.00089	0.26126	0.16157	0.11444	0.85625	0.16365	0.31106	0.12920	0.25453	*		
MEXICOAMERICANOS	0.22641	0.00604	0.63469	0.42125	0.36234	0.83437	0.05683	0.56668	0.01871	0.05603	0.51787	*	
ASIATICOS	0.22127	0.00624	0.66578	0.39184	0.32690	0.84675	0.04128	0.59558	0.00921	0.04485	0.43025	0.99990	*

Table S44. rs5030792 (TNFRSF1B) pairwise Fst

	MAZATECAS	PUREPECHAS	TARAHUMARAS	NAHUAS	MAYAS	TZELTALES	LACANDONES	HUICHOLES	MESTIZOS_JALISCO	EUROPEO	AFRICANO	MEXICOAMERICANOS
MAZATECAS	0.00000											
PUREPECHAS	0.00414	0.00000										
TARAHUMARAS	0.11521	0.08902	0.00000									
NAHUAS	0.23521	0.22553	0.04447	0.00000								

**MAYAS**	0.33737	0.33688	0.11290	0.00354	0.00000							
**TZELTALES**	0.00000	-0.00154	0.09916	0.21239	0.30502	0.00000						
**LACANDONES**	0.14729	0.11739	-0.01343	0.02835	0.08812	0.12473	0.00000					
**HUICHOLES**	0.14729	0.11739	-0.01343	0.02835	0.08812	0.12473	-0.01695	0.00000				
**MESTIZOS_JALISCO**	0.02820	0.00766	0.04276	0.20434	0.32868	0.02277	0.06030	0.06030	0.00000			
**EUROPEO**	0.00399	-0.00868	0.11482	0.28142	0.41296	-0.00070	0.15223	0.15223	0.01137	0.00000		
**AFRICANO**	-0.00810	0.00437	0.18038	0.34300	0.48162	-0.01201	0.23891	0.23891	0.03686	0.00537	0.00000	
**MEXICOAMERICANOS**	0.02041	-0.00215	0.05873	0.20572	0.32183	0.01417	0.08016	0.08016	-0.00343	0.00076	0.02854	0.00000

Table S45. rs5030792 (TNFRSF1B) Fst p values

	**MAZATECAS**	**PUREPECHAS**	**TARAHUMARAS**	**NAHUAS**	**MAYAS**	**TZELTALES**	**LACANDONES**	**HUICHOLES**	**MESTIZOS_JALISCO**	**EUROPEO**	**AFRICANO**	**MEXICOAMERICANOS**
**MAZATECAS**	*											
**PUREPECHAS**	0.52074	*										
**TARAHUMARAS**	0.02208	0.01960	*									
**NAHUAS**	0.00000	0.00000	0.07178	*								
**MAYAS**	0.00000	0.00000	0.00653	0.36046	*							
**TZELTALES**	0.99990	0.55994	0.06702	0.00050	0.00000	*						
**LACANDONES**	0.00248	0.00307	0.85447	0.11197	0.01564	0.01564	*					
**HUICHOLES**	0.00079	0.00129	0.84576	0.10553	0.01228	0.00911	0.99990	*				
**MESTIZOS_JALISCO**	0.11969	0.21839	0.03920	0.00000	0.00000	0.18711	0.01832	0.01663	*			
**EUROPEO**	0.57460	0.99990	0.00228	0.00000	0.00000	0.58717	0.00040	0.00000	0.12246	*		
**AFRICANO**	0.99990	0.55321	0.00059	0.00000	0.00000	0.99990	0.00000	0.00000	0.00564	0.20899	*	
**MEXICOAMERICANOS**	0.17117	0.47639	0.02851	0.00000	0.00000	0.33561	0.01267	0.00535	0.66815	0.32581	0.04356	*

Table S46 GLOBAL pairwise Fst

	MAZATECAS	PUREPECHAS	TARAHUMARAS	NAHUAS	MAYAS	TZELTALES	LACANDONES	HUICHOLES	MESTIZOS_JALISCO	EUROPEO	AFRICANO	MEXICOAMERICANOS	ASIATICOS
MAZATECAS	0.00000												
PUREPECHAS	0.06754	0.00000											
TARAHUMARAS	0.05885	0.00341	0.00000										
NAHUAS	0.03941	-0.00092	0.00649	0.00000									
MAYAS	0.02824	0.00639	-0.00427	0.01083	0.00000								
TZELTALES	0.04252	0.00461	-0.00390	0.00812	-0.01302	0.00000							
LACANDONES	0.01145	0.08574	0.06204	0.04984	0.03300	0.03025	0.00000						
HUICHOLES	0.01051	0.03326	0.03868	0.00357	0.02710	0.02547	0.02542	0.00000					
MESTIZOS_JALISCO	0.03145	0.04301	0.02297	0.02124	0.01319	0.00849	0.02628	0.01611	0.00000				
EUROPEO	0.08384	0.09895	0.08796	0.08450	0.07011	0.06522	0.09109	0.07249	0.04369	0.00000			
AFRICANO	0.27738	0.25112	0.23284	0.23861	0.23235	0.22110	0.29991	0.23754	0.17785	0.10007	0.00000		
MEXICOAMERICANOS	0.03174	0.09119	0.06893	0.06552	0.04662	0.05413	0.04467	0.03618	0.02298	0.03626	0.17588	0.00000	
ASIATICOS	0.18682	0.22266	0.18361	0.19343	0.17546	0.17238	0.20161	0.16414	0.12056	0.14962	0.15063	0.09277	0.00000

Table S47 GLOBAL Fst p values

	MAZATECAS	PUREPECHAS	TARAHUMARAS	NAHUAS	MAYAS	TZELTALES	LACANDONES	HUICHOLES	MESTIZOS_JALISCO	EUROPEO	AFRICANO	MEXICOAMERICANOS	ASIATIC
MAZATECAS	*												
PUREPECHAS	0.00000+-0.0000	*											
TARAHUMARAS	0.00000+-0.0000	0.11375+-0.0030	*										
NAHUAS	0.00000+-0.0000	0.29631+-0.0046	0.07494+-0.0027	*									
MAYAS	0.00178+-0.0004	0.06395+-0.0026	0.59054+-0.0052	0.03406+-0.0020	*								

**TZELTALES**	0.00089+-0.0003	0.12929+-0.0039	0.48490+-0.0044	0.10474+-0.0029	0.87575+-0.0033	*							
**LACANDONES**	0.04049+-0.0022	0.00000+-0.0000	0.00000+-0.0000	0.00000+-0.0000	0.00040+-0.0002	0.01020+-0.0010	*						
**HUICHOLES**	0.03663+-0.0019	0.00000+-0.0000	0.00020+-0.0001	0.15068+-0.0035	0.00158+-0.0004	0.01089+-0.0011	0.00198+-0.0005	*					
**MESTIZOS_JALISCO**	0.00000+-0.0000	0.00000+-0.0000	0.00000+-0.0000	0.00000+-0.0000	0.00446+-0.0007	0.06465+-0.0022	0.00020+-0.0001	0.00366+-0.0006	*				
**EUROPEO**	0.00000+-0.0000	0.00000+-0.0000	0.00000+-0.0000	0.00000+-0.0000	0.00000+-0.0000	0.00000+-0.0000	0.00000+-0.0000	0.00000+-0.0000	0.00000+-0.0000	*			
**AFRICANO**	0.00000+-0.0000	0.00000+-0.0000	0.00000+-0.0000	0.00000+-0.0000	0.00000+-0.0000	0.00000+-0.0000	0.00000+-0.0000	0.00000+-0.0000	0.00000+-0.0000	0.00000+-0.0000	*		
**MEXICOAMERICANOS**	0.00010+-0.0001	0.00000+-0.0000	0.00000+-0.0000	0.00000+-0.0000	0.00000+-0.0000	0.00010+-0.0001	0.00000+-0.0000	0.00030+-0.0002	0.00010+-0.0001	0.00000+-0.0000	0.00000+-0.0000	*	
**ASIATICOS**	0.00000+-0.0000	0.00000+-0.0000	0.00000+-0.0000	0.00000+-0.0000	0.00000+-0.0000	0.00000+-0.0000	0.00000+-0.0000	0.00000+-0.0000	0.00000+-0.0000	0.00000+-0.0000	0.00000+-0.0000	0.00000+-0.0000	*

Table S48. Distances Fst (down) p values (up)

	MAZATECAS	PUREPECHAS	TARAHUMARAS	NAHUAS	MAYAS	TZELTALES	LACANDONES	HUICHOLES	MESTIZOS_JALISCO	EUROPEO	AFRICANO	MEXICOAMERICANOS	ASIATICOS
**MAZATECAS**	0.00000	0.00000	0.00000	0.00000	0.00178	0.00089	0.04049	0.03663	0.00000	0.00000	0.00000	0.00010	0.00000
**PUREPECHAS**	0.06754	0.00000	0.11375	0.29631	0.06395	0.12929	0.00000	0.00000	0.00000	0.00000	0.00000	0.00000	0.00000
**TARAHUMARAS**	0.05885	0.00341	0.00000	0.07494	0.59054	0.48490	0.00000	0.00020	0.00000	0.00000	0.00000	0.00000	0.00000
**NAHUAS**	0.03941	-0.00092	0.00649	0.00000	0.03406	0.10474	0.00000	0.15068	0.00000	0.00000	0.00000	0.00000	0.00000
**MAYAS**	0.02824	0.00639	-0.00427	0.01083	0.00000	0.87575	0.00040	0.00158	0.00446	0.00000	0.00000	0.00000	0.00000
**TZELTALES**	0.04252	0.00461	-0.00390	0.00812	-0.01302	0.00000	0.01020	0.01089	0.06465	0.00000	0.00000	0.00010	0.00000
**LACANDONES**	0.01145	0.08574	0.06204	0.04984	0.03300	0.03025	0.00000	0.00198	0.00020	0.00000	0.00000	0.00000	0.00000
**HUICHOLES**	0.01051	0.03326	0.03868	0.00357	0.02710	0.02547	0.02542	0.00000	0.00366	0.00000	0.00000	0.00030	0.00000
**MESTIZOS_JALISCO**	0.03145	0.04301	0.02297	0.02124	0.01319	0.00849	0.02628	0.01611	0.00000	0.00000	0.00000	0.00010	0.00000
**EUROPEO**	0.08384	0.09895	0.08796	0.08450	0.07011	0.06522	0.09109	0.07249	0.04369	0.00000	0.00000	0.00000	0.00000
**AFRICANO**	0.27738	0.25112	0.23284	0.23861	0.23235	0.22110	0.29991	0.23754	0.17785	0.10007	0.00000	0.00000	0.00000
**MEXICOAMERICANOS**	0.03174	0.09119	0.06893	0.06552	0.04662	0.05413	0.04467	0.03618	0.02298	0.03626	0.17588	0.00000	0.00000

**ASIATICOS**	0.18682	0.22266	0.18361	0.19343	0.17546	0.17238	0.20161	0.16414	0.12056	0.14962	0.15063	0.09277	0.00000

Table S49. Linkage disequilibrium analysis by variation and between variants in all populations:

**POPULATION MAZATECAS**<br>Exact tests for linkage		
	P	**Locus combination**
	0.006250	BsmI/TaqI
	0.000000	BsmI/CTR
	0.002500	ApaI/CTR
	0.000000	TaqI/CTR
	0.000000	COL1A1/CTR
	0.000000	XbaI/CTR
	0.000000	CYP19/CTR
	0.007812	BGP/CTR
	0.000313	CTR/TGFBI
	0.000000	CTR/IL6
	0.000000	CTR/PTHRI
	0.000000	CTR/OPG950
	0.000937	CTR/TNF593
	0.000000	CTR/TNF598
	0.000000	CTR/TNF620

Table S50. Linkage disequilibrium analysis by variation and between variants in all populations:

**POPULATION PURHEPECHAS**<br>Exact tests for linkage		
	P	**Locus combination**
	0.000000	BsmI/CTR
	0.035937	BsmI/TNF593
	0.000000	ApaI/CTR
	0.003750	ApaI/PTHRI
	0.027813	ApaI/TNF593
	0.000000	TaqI/CTR
	0.032813	TaqI/TNF593
	0.000000	COL1A1/CTR
	0.009375	COL1A1/TNF593
	0.000000	XbaI/CTR
	0.036250	XbaI/TGFBI
	0.000000	CYP19/CTR
	0.019688	CYP19/TNF593
	0.000000	BGP/CTR
	0.031875	BGP/TGFBI
	0.022500	BGP/TNF593
	0.000000	CTR/IL6
	0.000313	CTR/PTHRI
	0.000313	CTR/OPG950
	0.000000	CTR/TNF593
	0.000000	CTR/TNF598
	0.000000	CTR/TNF620
	0.046250	TGFBI/OPG950
	0.003438	TGFBI/TNF593
	0.036875	TGFBI/TNF620

	0.005000	IL6/TNF593
	0.023125	OPG950/TNF593
	0.004687	TNF593/TNF598

Table S51. Linkage disequilibrium analysis by variation and between variants in all populations:

**POPULATION TARAHUMARAS**<br>Exact tests for linkage		
	P	**Locus combination**
	0.003125	ApaI/CTR
	0.000000	ApaI/TNF598
	0.004063	TaqI/CTR
	0.044375	TaqI/TGFBI
	0.000313	TaqI/TNF598
	0.013750	COL1A1/CYP19
	0.000313	COL1A1/CTR
	0.000000	COL1A1/TNF598
	0.027813	XbaI/CYP19
	0.005000	XbaI/CTR
	0.000625	XbaI/TNF598
	0.000625	CYP19/CTR
	0.000313	CYP19/TNF598
	0.034063	BGP/CTR
	0.011875	BGP/TNF598
	0.000625	CTR/TGFBI
	0.000000	CTR/IL6
	0.004063	CTR/PTHRI
	0.000313	CTR/OPG950

	0.010000	CTR/TNF593
	0.000000	CTR/TNF598
	0.005625	CTR/TNF620
	0.000000	TGFBI/TNF598
	0.000000	IL6/TNF598
	0.007188	PTHRI/TNF598
	0.000000	OPG950/TNF598
	0.000000	TNF593/TNF598
	0.002500	TNF598/TNF620

Table S52. Linkage disequilibrium analysis by variation and between variants in all populations:

POPULATION NAHUAS		
	0.000313	BsmI/TaqI
	0.008438	BsmI/COL1A1
	0.023125	BsmI/XbaI
	0.000000	BsmI/CTR
	0.004063	ApaI/TaqI
	0.000313	ApaI/CTR
	0.004063	TaqI/COL1A1
	0.012500	TaqI/XbaI
	0.016563	TaqI/CYP19

	0.015938	TaqI/BGP
	0.000000	TaqI/CTR
	0.035312	TaqI/TGFBI
	0.013750	TaqI/IL6
	0.013125	TaqI/TNF593
	0.000625	TaqI/TNF598
	0.041250	COL1A1/XbaI
	0.000000	COL1A1/CTR
	0.000000	CTR/TGFBI
	0.000000	CTR/IL6
	0.000625	CTR/PTHRI
	0.000000	CTR/OPG950
	0.000625	CTR/TNF593
	0.000313	CTR/TNF598
	0.000000	CTR/TNF620
	0.000313	TNF593/TNF598

Table S53. Linkage disequilibrium analysis by variation and between variants in all populations:

**POPULATION MAYAS**<br>Exact tests for linkage		
	P	**Locus combination**
	0.001563	BsmI/TaqI
	0.000000	BsmI/CTR
	0.000313	ApaI/CTR
	0.016250	TaqI/BGP
	0.000000	TaqI/CTR
	0.007500	TaqI/TNF593
	0.035000	TaqI/OPG950
	0.000000	COL1A1/CTR
	0.000000	XbaI/CTR
	0.000000	CYP19/CTR
	0.000000	BGP/CTR
	0.040938	BGP/OPG950
	0.000000	CTR/TGFBI
	0.000000	CTR/IL6
	0.000000	CTR/PTHRI
	0.000000	CTR/OPG950
	0.000000	CTR/TNF593
	0.000000	CTR/TNF598
	0.000000	CTR/TNF620
	0.010937	OPG950/TNF593

Table S54. Linkage disequilibrium analysis by variation and between variants in all populations:

**POPULATION TZELTALES**<br>Exact tests for linkage		
	P	**Locus combination**
	0.040625	BsmI/ApaI
	0.015625	BsmI/TaqI
	0.000313	BsmI/CTR
	0.001563	BsmI/CYP19
	0.001563	ApaI/CYP19
	0.003750	ApaI/CTR
	0.000313	ApaI/OPG950
	0.000937	TaqI/CYP19
	0.001875	TaqI/CTR
	0.000625	COL1A1/CYP19
	0.000313	XbaI/CYP19
	0.001563	XbaI/CTR
	0.000625	CYP19/BGP
	0.000000	CYP19/CTR
	0.000000	CYP19/TGFBI
	0.000000	CYP19/IL6
	0.000313	CYP19/PTHRI
	0.000625	CYP19/OPG950
	0.000625	CYP19/TNF593
	0.000000	CYP19/TNF598
	0. 001250	CYP19/TNF620
	0.000000	BGP/CTR
	0.003438	CTR/TGFBI
	0.000313	CTR/IL6

	0.004063	CTR/PTHRI
	0.001563	CTR/OPG950
	0.008125	CTR/TNF593
	0.000000	CTR/TNF598
	0.010937	CTR/TNF620
	0.022187	PTHRI/OPG950

Table S55. Linkage disequilibrium analysis by variation and between variants in all populations:

**POPULATION LACANDONES**<br>Exact tests for linkage		
	P	**Locus combination**
	0.000000	BsmI/CTR
	0.000937	BsmI/TGFBI
	0.000000	ApaI/CTR
	0.000000	TaqI/CTR
	0.000625	TaqI/TGFBI
	0.000000	COL1A1/CTR
	0.001250	COL1A1/TGFBI
	0.017500	XbaI/BGP
	0.000000	XbaI/CTR
	0.024375	XbaI/TGFBI
	0.000000	CYP19/CTR
	0.002188	CYP19/TGFBI
	0.000000	BGP/CTR
	0.000000	BGP/TGFBI
	0.000000	CTR/TGFBI

	0.000000	CTR/IL6
	0.000000	CTR/PTHRI
	0.000000	CTR/OPG950
	0.000000	CTR/TNF593
	0.000000	CTR/TNF598
	0.000000	CTR/TNF620
	0.001875	TGFBI/IL6
	0.025937	TGFBI/TNF593
	0.000625	TGFBI/TNF598
	0.006875	TGFBI/TNF620
	0.414062	IL6/PTHRI

Table S56. Linkage disequilibrium analysis by variation and between variants in all populations:

**POPULATION HUICHOLES**<br>Exact tests for linkage		
	P	**Locus combination**
	0.007500	BsmI/CTR
	0.036875	ApaI/TaqI
	0.005000	ApaI/COL1A1
	0.009062	ApaI/CYP19
	0.036562	ApaI/BGP
	0.000625	ApaI/CTR
	0.005938	ApaI/IL6
	0.014063	ApaI/TNF593
	0.000625	ApaI/TNF620
	0.001563	TaqI/CTR
	0.038125	TaqI/TNF620
	0.000000	COL1A1/CTR

	0.013437	COL1A1/TNF620
	0.006875	XbaI/CTR
	0.023125	XbaI/TNF620
	0.000000	CYP19/CTR
	0.012500	CYP19/TNF620
	0.005000	BGP/CTR
	0.011250	BGP/TNF620
	0.006875	CTR/TGFBI
	0.000000	CTR/IL6
	0.016250	CTR/PTHRI
	0.018750	CTR/OPG950
	0.000625	CTR/TNF593
	0.000937	CTR/TNF598
	0.000000	CTR/TNF620
	0.012812	TGFBI/TNF620
	0.011562	IL6/TNF620
	0.020938	PTHRI/OPG950
	0.037812	PTHRI/TNF593
	0.019688	PTHRI/TNF620
	0.019688	OPG950/TNF620
	0.019688	TNF593/TNF620
	0.029375	TNF598/TNF620

Table S57. Linkage disequilibrium analysis by variation and between variants in all populations:

**POPULATION MESTIZOS**<br>Exact tests for linkage		
	P	**Locus combination**
	0.000000	BsmI/ApaI

	0.000000	BsmI/TaqI
	0.000000	BsmI/COL1A1
	0.000000	BsmI/XbaI
	0.000000	BsmI/CYP19
	0.000000	BsmI/BGP
	0.000000	BsmI/CTR
	0.000000	BsmI/TGFBI
	0.000000	BsmI/IL6
	0.000000	BsmI/PTHRI
	0.000000	BsmI/OPG950
	0.000000	BsmI/TNF593
	0.000000	BsmI/TNF598
	0.000000	BsmI/TNF620
	0.000000	ApaI/TaqI
	0.013750	ApaI/XbaI
	0.000000	ApaI/CTR
	0.001250	ApaI/TGFBI
	0.000000	TaqI/CTR
	0.002188	TaqI/TGFBI
	0.002812	COL1A1/XbaI
	0.000000	COL1A1/CTR
	0.004063	COL1A1/TGFBI
	0.028438	XbaI/BGP
	0.000000	XbaI/CTR
	0.000000	XbaI/TGFBI
	0.000000	XbaI/IL6
	0.021562	XbaI/PTHRI
	0.020625	XbaI/OPG950
	0.010313	XbaI/TNF593
	0.005000	XbaI/TNF598
	0.005938	XbaI/TNF620

	0.000000	CYP19/CTR
	0.004375	CYP19/TGFBI
	0.000000	BGP/CTR
	0.031250	BGP/TGFBI
	0.000000	CTR/TGFBI
	0.000000	CTR/IL6
	0.000000	CTR/PTHRI
	0.000000	CTR/OPG950
	0.000000	CTR/TNF593
	0.000000	CTR/TNF598
	0.000000	CTR/TNF620
	0.000000	TGFBI/IL6
	0.021875	TGFBI/PTHRI
	0.001563	TGFBI/OPG950
	0.016563	TGFBI/TNF593
	0.000313	TGFBI/TNF598
	0.014063	TGFBI/TNF620
	0.003125	TNF593/TNF620

Figure S1. Linkage disequilibrium map and block:

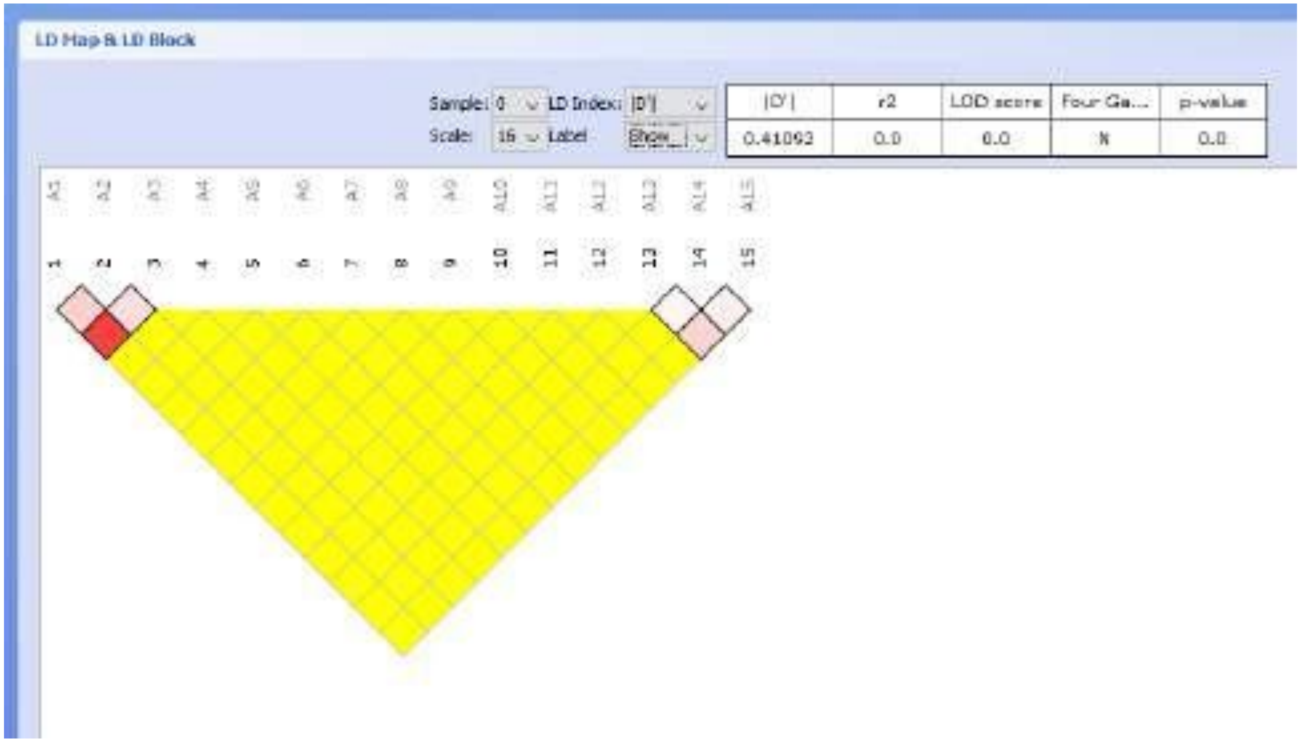

1 D'= 0.42307

2 D'= 0.86679

3 D'= 0.35894

13 D'= 0.20558

14 D'= 0.41092

15 D’= 0.24963
